# Horizontal acquisition of a hypoxia-responsive molybdenum cofactor biosynthesis pathway contributed to *Mycobacterium tuberculosis* pathoadaptation

**DOI:** 10.1371/journal.ppat.1006752

**Published:** 2017-11-27

**Authors:** Florence Levillain, Yannick Poquet, Ludovic Mallet, Serge Mazères, Michael Marceau, Roland Brosch, Franz-Christoph Bange, Philip Supply, Axel Magalon, Olivier Neyrolles

**Affiliations:** 1 Institut de Pharmacologie et de Biologie Structurale, Université de Toulouse, CNRS, UPS, Toulouse, France; 2 Unité de Mathématique et Informatique Appliquées de Toulouse, INRA, UR875, Castanet-Tolosan, France; 3 Univ. Lille, CNRS, Inserm, CHU Lille, Institut Pasteur de Lille, U1019 - UMR 8204 - CIIL - Centre d’Infection et d’Immunité de Lille, Lille, France; 4 Institut Pasteur, Unit for Integrated Mycobacterial Pathogenomics, Paris, France; 5 Department of Medical Microbiology and Hospital Epidemiology, Hannover Medical School, Hannover, Germany; 6 Aix-Marseille University, CNRS, IMM, LCB UMR7283, Marseille, France; McGill UniversityHealth Centre, CANADA

## Abstract

The unique ability of the tuberculosis (TB) bacillus, *Mycobacterium tuberculosis*, to persist for long periods of time in lung hypoxic lesions chiefly contributes to the global burden of latent TB. We and others previously reported that the *M*. *tuberculosis* ancestor underwent massive episodes of horizontal gene transfer (HGT), mostly from environmental species. Here, we sought to explore whether such ancient HGT played a part in *M*. *tuberculosis* evolution towards pathogenicity. We were interested by a HGT-acquired *M*. *tuberculosis*-specific gene set, namely *moaA1-D1*, which is involved in the biosynthesis of the molybdenum cofactor. Horizontal acquisition of this gene set was striking because homologues of these *moa* genes are present all across the *Mycobacterium* genus, including in *M*. *tuberculosis*. Here, we discovered that, unlike their paralogues, the *moaA1-D1* genes are strongly induced under hypoxia. *In vitro*, a *M*. *tuberculosis moaA1-D1*-null mutant has an impaired ability to respire nitrate, to enter dormancy and to survive in oxygen-limiting conditions. Conversely, heterologous expression of *moaA1-D1* in the phylogenetically closest non-TB mycobacterium, *Mycobacterium kansasii*, which lacks these genes, improves its capacity to respire nitrate and grants it with a marked ability to survive oxygen depletion. *In vivo*, the *M*. *tuberculosis moaA1-D1*-null mutant shows impaired survival in hypoxic granulomas in C3HeB/FeJ mice, but not in normoxic lesions in C57BL/6 animals. Collectively, our results identify a novel pathway required for *M*. *tuberculosis* resistance to host-imposed stress, namely hypoxia, and provide evidence that ancient HGT bolstered *M*. *tuberculosis* evolution from an environmental species towards a pervasive human-adapted pathogen.

## Author summary

*Mycobacterium tuberculosis*, the etiological agent of tuberculosis (TB), can persist for years and even decades in the lungs of its human host. Here we report that a unique *M*. *tuberculosis* gene cluster involved in the synthesis of the molybdenum cofactor, a cofactor for several oxidoreductases including the nitrate reductase, allows this major pathogen to respire nitrate and to persist in a dormant state under hypoxia, a stress condition encountered in lung TB lesions. Strikingly the *M*. *tuberculosis* ancestor, which most likely was an environmental harmless bacterium, acquired this gene cluster, together with its hypoxia-responsive transcriptional regulator, horizontally from neighboring bacteria. Our results uncover a key step in *M*. *tuberculosis* evolution towards pathogenicity.

## Introduction

*Mycobacterium tuberculosis* (*Mtb*) is an obligate, strictly human-adapted pathogen of major public health importance [1]. One of the most striking features of this microorganism is its ability to persist in lung lesions, the granulomas, for years and even decades in a so-called “dormant” state, before it eventually reactivates and causes tuberculosis (TB) disease. Up to one fourth of the global population is thought to carry such latent *Mtb* infection [2]. The human granuloma is an acidic, nutrient-poor and highly hypoxic environment [3]. To survive such hostile conditions, *Mtb* is thought to have evolved multiple metabolic mechanisms, including the use of fatty acids as carbon and energy sources for example [4]. How these mechanisms were acquired remains largely unknown.

Horizontal gene transfer (HGT) is a primary driving force in evolution of prokaryotes [5]. By enabling the sudden acquisition of novel metabolic functions, HGT can shift the overall ecology of bacterial recipients, granting them with the ability to colonize new environments, including new living hosts, in a pathogenic or non-pathogenic (*i*.*e*. symbiotic or commensal) manner [6,7]. The recent evolution of *Mtb* and related species of the so-called *Mtb* complex (MTBC), which cause TB in human and other mammals, is considered mostly clonal, with few if any HGT events detected within the complex or between the complex and other species [8]. However comparative genomics and other *in silico* studies have revealed important episodes of HGT in early steps after the divergence of non-tuberculous mycobacterial species, including in branches of TB bacilli represented by *Mycobacterium canettii* (also known as smooth tubercle bacilli, STB) that preceded the emergence of the MTBC ancestor [8–14]. Many of these exogenously acquired genes are clustered into genomic islands, and the use of parametric methods allowed us to assign phylogenetic origins to most of them. Strikingly, a large fraction of these genes were acquired from environmental species, belonging to the genera Rhizobiales, Pseudomonadales, Burkholderiales, and Bifidobacteriales [9,10], supporting the previous assumption that the MTBC ancestor was most likely an environmental species [15].

Here, we explored whether these ancient episodes of HGT played a role in the acquisition of virulence mechanisms during *Mtb* evolution, rather than reflecting natural gene flow. We were particularly interested by a 15-kb genomic island, *rv3108-26c* [15], hereinafter named Moco-1, which contains genes, namely *moaA1-D1*, organized into an operon and predicted to encode enzymes involved in the early steps of the molybdenum cofactor (Moco) biosynthesis pathway [16,17]. In all living organisms, Moco and related Mo-containing cofactors are required for the proper function of various oxidoreductases, including the canonical prokaryotic nitrate reductase (NR) NarGHI [16,18], which fulfills both nitrate assimilatory and anaerobic respiratory functions in *Mtb* [19–22]. The *moaA1-D1* gene set was previously reported to be required for NR-mediated nitrate assimilation by *Mtb* when bacteria are grown on nitrate as sole nitrogen source under aerobic conditions [23]. Whether these genes are required for nitrate respiration under hypoxia has not been investigated. The exclusive presence of Moco-1 in genomes of TB-causing mycobacteria strikingly contrasts with the universal distribution of homologues of these genes, namely *moaA2-D2*, among *Mtb* and all other mycobacteria [17]. Thus we wondered whether acquisition of the supplemental *moaA1-D1* gene set influenced *Mtb* pathoadaptation.

In this article, we show that the horizontally acquired *moaA1-D1* genes, but not their vertically transmitted *moaA2-D2* homologues, are up-regulated by the hypoxia-inducible transcriptional regulator MoaR1, which also belongs to the Moco-1 genomic island. Our data show that hypoxia-specific induction of the *moaA1-D1* locus sustains *Mtb* nitrate respiration and persistence in the absence of oxygen *in vitro* and *in vivo*. Altogether, our study identifies a novel pathway required for *Mtb* resistance to hypoxia, and uncovers a key contribution of ancient HGT to the evolutionary success of this major pathogen.

## Results

### Evolutionary history of the *Mtb* Moco-1 genomic island and other *moa* genes

The organization of the *moa* and related genes involved in the Moco biosynthesis pathway (S1 Fig) is very complex in *Mtb*, with essentially three sets of genes numbered as one to three [15,23].

**Fig 1:**
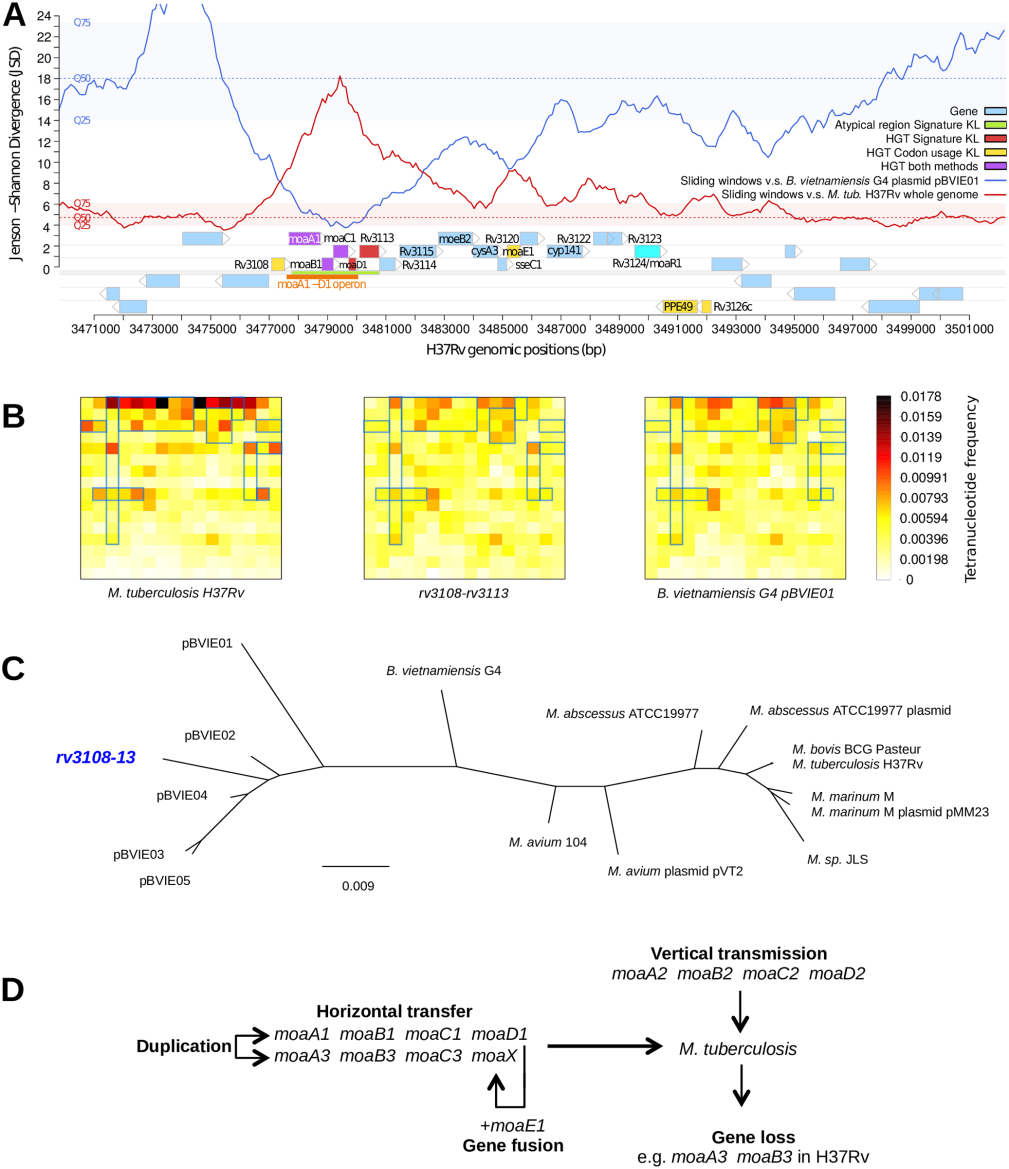
The *Mtb moaA1-D1* locus has been acquired by horizontal gene transfer. (A) Jensen-Shannon divergence profiles of the *Mtb* genomic region harboring the Moco-1 island and its genomic context. The red curve represents the divergence between the signature of the whole *Mtb* genome and that of the sliding windows (5-kb long, 100-bp step) over the region. The blue curve represents the divergence between the signature of the *B*. *vietnamiensis* G4 plasmid pBVIE01 and that of the sliding windows over the region. The distribution of the whole *Mtb* genome windows divergence compared to either signature profile is provided to help interpret the local variations, with the medians in dashed lines (Mtb in red, pBVIE01 in blue) and color-filled strips (Mtb in red, pBVIE01 in blue) delimiting the first (Q25) and third (Q75) quartiles of the distributions. Regular genes are displayed in light blue. Genes in red, yellow and purple were identified as incongruent with the whole *Mtb* genome based on genomic signature analysis [9,24], atypical codon usage [54] or both, respectively. These computations were performed using the GOHTAM program [25]. The genomic region in green was found atypical in genomic signature analysis [9,24]. The *moaA1-D1* operon is indicated by the orange strip; the transcriptional activator-encoding gene *rv3124*/*moaR1* [35] is indicated in cyan. Importantly, the divergence of the *rv3108-13* cluster to pBVIE01 locally falls to values that can be described as “intragenomic variability” (red-colored strip for *Mtb*). These values are usually observed when comparing a genome signature to its own sliding windows. (B) Compositional profile (Chaos game representation of tetranucleotide frequencies [59]) of *Mtb* H37Rv (left panel), the *rv3108-13* locus (central panel) and the *B*. *vietnamiensis* G4 pBVIE01 plasmid (right panel). Heatmap matrixes represent the frequencies of the 256 tetranucleotides for each sequence. Visually similar heatmaps suggest highly similar tetranucleotide composition. Blue rectangles indicate subpatterns that clearly cluster the *rv3108-13* locus with pBVIE01. (C) Tetranucleotide profile similarity neighbor-joining tree. The distances (Euclidean) and scale are in arbitrary units. The *Mtb rv3108-13* locus is shown in blue, strikingly clustering better with *B*. *vietnamiensis* plasmids than with its host genome and other mycobacterial genomes. (D) The complex organization of *moa* genes in *Mtb* likely results from a combination of vertical transmission (*moaA2-D2*), horizontal gene transfer (*moaA1-D1*), gene duplication (*moaA3-X*) and fusion (*moaX*), and gene loss (*moaA3*,*B3* in H37Rv).

The *moaA1-D1* genes, which are involved in the early steps of the Moco biosynthesis pathway (S1 Fig), are embedded in a ~15-kb genome fragment, corresponding to the *rv3108-26c* genes and hereinafter named Moco-1. We and others previously identified Moco-1 as a genomic island acquired through HGT after the divergence from the phylogenetically closest non-tuberculous mycobacterial species, *Mycobacterium marinum* and *Mycobacterium kansasii* [9,11,12]. In particular, the genomic signature pattern, *i*.*e*. the tetranucleotide frequency [24], of the first ~4-kb fragment of Moco-1, *i*.*e*. *rv3108-13*, which includes the *moaA1-D1* genes, is incongruent with that of the *Mtb* genome as a whole (Fig 1A–1C). This suggests that i/ Moco-1 is a mosaic resulting from two or more HGT events, or that ii/ Moco-1 was acquired in a single HGT event followed by compositional amelioration/erosion of the rest of the island, *i*.*e*. *rv3114-26c* (Fig 1A). A genomic signature-based phylogenetic analysis using the Genomic Origin of Horizontal Transfers and Metagenomics (GOHTAM) program [25] indicated that the *rv3108-13* locus clusters with plasmids of the β-proteobacterium species *Burkholderia vietnamiensis* (Fig 1A–1C), suggesting a possible origin for Moco-1.

The Moco-1 region was examined in *M*. *canettii* isolates, obtained from human TB cases but thought to represent earlier stages of pathoadaptive evolution relative to the MTBC (8, 14, 45, 46). Strikingly, the Moco-1 region was detected as a complete genomic island only in some *M*. *canettii* strains, including representatives of the genomically most divergent lineages from the MTBC (*i*.*e*. STB-K, -J, and -I; S2 Fig), suggesting that the Moco-1 region was already acquired by the common progenitor of *M*. *canettii*. In other *M*. *canettii* strains, such as STB-A, -D, -E, -H and -L, a sequence block from the *rv3112* to *rv3125c* orthologues is missing, resulting in the truncation of the *rv3111* (*moaC1*) and *rv3126c* orthologues on both flanks, with an identical junction sequence among the strains (S2 Fig). The latter observation suggests that the Moco-1 region was partially deleted in a single event at a common node during subsequent divergence of some *M*. *canettii* lineages. However, it is also possible that the region was first acquired and/or partially deleted in individual lineages, and then shuttled to other lineages by intra-species recombination. Indeed, we inferred traces of such shuttling between *M*. *canettii* strains from the observation of an almost perfect sequence match between gene blocks from at least *rv3105c* to *rv3117* orthologues of STB-I and STB-G, contrasting with the much higher SNP density detected in neighboring regions between both strains (S3 Fig).

A second *moa* gene cluster, namely *moaA3*-*moaB3*-*moaC3*-*moaX* (in which *moaX* results from a *moaD-E* gene fusion [23]), is also present both in *M*. *canettii* and MTBC genomes but not in other mycobacteria (Fig 1D, S4 Fig), and was thus proposed to also result from ancient HGT into a common ancestor of the TB bacilli [11]. The close sequence similarity between these genes and the *moaA1-D1* genes (S4 Fig) suggests that they originate from duplication from the *moaA1-D1* locus. However, they have been subject to partial gene loss at least in *Mtb* H37Rv, where part of *moaB3* and the entire *moaA3* genes are absent due to an IS*6110*-mediated deletion [26].

Finally, *Mtb* contains a third set of *moa* genes, namely *moaA2-D2*, which are present in all fast- and slow-growing *Mycobacterium* species that we examined, indicating vertical transmission from a distant, common mycobacterial ancestor (S4 Fig, Fig 1D). This latter gene cluster encompasses the unique adenylyltransferase-encoding gene *mog* (*rv0865*), which is needed for MoCo biosynthesis (S1 Fig) [16].

Thus, it appears that, relative to non-tuberculous mycobacteria, the architecture of the *moa* gene sets is original and quite plastic in the MTBC and in *M*. *canettii* strains, consisting of a complex combination of horizontally acquired (*moaA1-D1)*, vertically transmitted (*moaA2-D2*), and duplicated and diversified (*moaA3-X*) gene clusters. Some of them show post-HGT modifications such as partial gene loss, e.g. the absence of *moaA3* and *moaB3* from *Mtb* H37Rv or the absence of selected genes in the Moco-1 region from some *M*. *canettii* strains, as well as traces of gene fusion events, e.g. *moaX* in the MTBC. This led us to ask which had been the selective advantage, if any, of the horizontal acquisition of supplemental sets of *moa* genes by the TB bacilli during evolution. To address this question, we used the *Mtb* H37Rv strain, which contains only one set of HGT-acquired *moa* genes, *i*.*e*. *moaA1-D1*, but not the complete *moaA3-X* cluster, in addition to vertically transmitted *moa* genes, *i*.*e*. *moaA2-D2*.

### The *moaA1-D1* locus favors *Mtb* nitrate respiration and persistence under hypoxia

**Fig 2:**
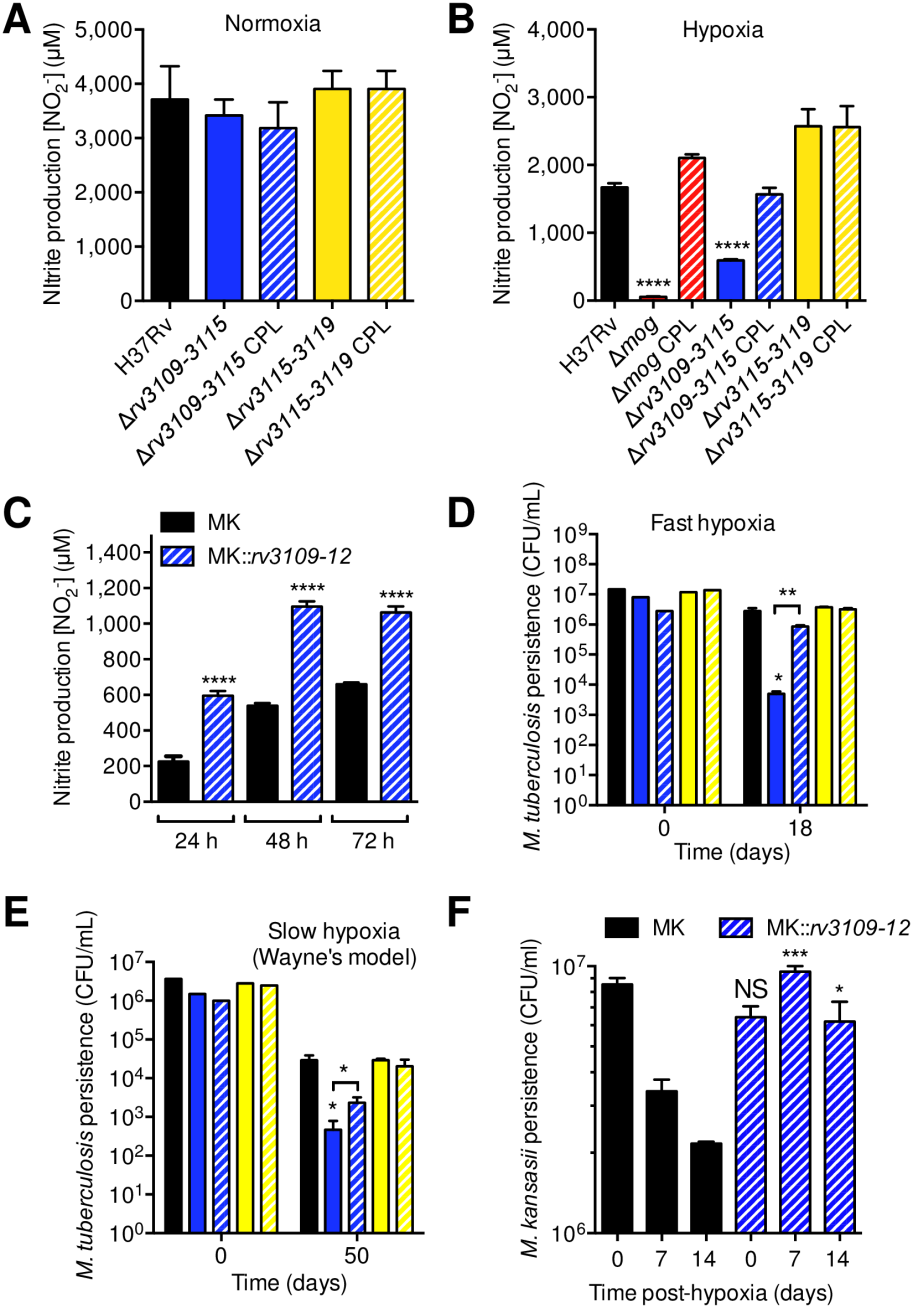
The *moaA1-D1* locus is required for *Mtb* nitrate respiration and survival to hypoxic stress. (A and B) Bacteria were cultivated for 14 days in modified Sauton’s minimal medium in normoxic (A) or hypoxic, after fast O_2_ depletion, conditions (B). Nitrate reduction was quantified by measuring nitrite production. (C) Utilization of nitrate by *M*. *kansasii* wild type (MK) or a recombinant MK expressing the *rv3109-12* locus (MK::*rv3109-12*). Bacteria were cultivated as in (B) after fast O_2_ depletion. (D and E) Bacteria were cultivated for 18 days in Dubos medium after fast O_2_ depletion (D), or for 50 days in the Wayne model (E). In (A,B,D,E), the Δ*rv3109-15* mutant was complemented with the I528 cosmid, and the Δ*rv3115-19* mutant was complemented with the IE240 cosmid. (F) Survival of *M*. *kansasii* wild type (MK) or a recombinant MK expressing the *rv3109-12* locus (MK::*rv3109-12*) under hypoxia. Bacteria were cultivated as in (D) after fast O_2_ depletion. In (D) and (E) bar colors are identical to those used in (A) and (B). In all panels, data show mean±s.e.m. of biological replicates (n≥3). Each biological replicate was measured in technical triplicates and statistical analyses were performed on the summary of all the biological replicates. Data were analyzed using the Student’s *t*-test; *, *P*<0.05; **, *P*<0.01; ****, *P*<0.0001. The graphs are representative of at least 3 independent experiments.

The *moaA1-D1* locus is known to be involved in nitrate assimilation when nitrate is provided as sole nitrogen source [23]. Here we asked whether this locus is required for nitrate respiration, *i*.*e*. to use nitrate as terminal electron acceptor, in the absence of oxygen. We generated a *Mtb* H37Rv mutant deleted of the *rv3109-15* locus (Δ*rv3109-15*), which encompasses the *moaA1-D1* operon, using the recombineering technology [27] and its corresponding complemented variant. In normoxic conditions (21% O_2_), the Δ*rv3109-15* mutant did not show any defect in nitrate reduction compared to the wild type and complemented strains, as measured by nitrite production in the culture medium (Fig 2A), and in line with previous findings [23]. In contrast, the Δ*rv3109-15* mutant was impaired in nitrate respiration under hypoxic conditions (<1% O_2_), and this phenotype was reversed by genetic complementation using an integrating cosmid carrying a genomic insert from Mtb H37Rv that over-spans the deleted region and flanking sections and provides gene expression from their natural promoters (Fig 2B). Similarly, transformation of the Δ*rv3109-15* mutant with an integrative plasmid expressing the *moaA1-D1* genes from a strong, constitutive promoter also led to a complemented phenotype (S5 Fig), This latter finding confirmed the importance of *moaA1-D1* for sustaining nitrate respiration under hypoxia and validated results obtained by the cosmid-complemented construct. For the remaining experiments in this study, the cosmid-complemented construct was used. As a control, a mutant deleted in the *mog* gene (Δ*mog*), in which Moco biosynthesis is supposed to be abolished (S1 Fig), was totally impaired in its ability to reduce nitrate in hypoxic conditions, which was also reversed after genetic complementation (Fig 2B). In order to extend our finding to other parts of the Moco-1 genomic island, we generated a mutant deleted of the *rv3115-19* locus (Δ*rv3115-19*), which contains the *moeB2* and *moaE1* genes. This mutant did not show any defect in nitrate reduction, neither in normoxic nor in hypoxic conditions (Fig 2A and 2B). These results demonstrate that within Moco-1, the *moaA1-D1* locus plays a key part in proper function of the *Mtb* NR, at least in hypoxic conditions; under these conditions, the *moeB2* and *moaE1* genes are dispensable, either because they are not functional or because their function is redundant with that of their *moeB1* and *moaE2* homologues. In a reverse evolution experiment, we also found that heterologous overexpression of *moaA1-D1* in *M*. *kansasii*, a species close to the MTBC but which does not harbor the *moaA1-D1* locus, resulted in an enhanced ability of this bacterium to reduce nitrate (Fig 2C), further strengthening our conclusion on the role of *moaA1-D1* in nitrate reduction.

Because nitrate reduction sustains *Mtb* persistence under hypoxia *in vitro* [19], we asked whether the impaired ability of the *Mtb* Δ*rv3109-15* mutant to reduce nitrate had any consequences on its capacity to survive hypoxic stress. In two models of rapid and slow (the “Wayne model” [28], which allows studying later events during hypoxia and dormancy) O_2_ depletion, the Δ*rv3109-15* mutant showed a defect in surviving hypoxic stress by 3- and 2-orders of magnitude, respectively, which was reversed upon genetic complementation (Fig 2D and 2E). Interestingly, genetic deletion of the 3’-fragment of the Moco-1 island in the Δ*rv3115-19* mutant did not affect the ability of *Mtb* to survive slow or fast hypoxic stress (Fig 2D and 2E). Importantly and in line with our results in *Mtb*, heterologous expression of *moaA1-D1* in *M*. *kansasii* enabled this bacterium to survive hypoxia, while wild-type *M*. *kansasii* progressively dies in this condition (Fig 2F). Altogether, these data show that although *moaA1-D1* is dispensable for nitrate reduction in normoxic conditions, this locus is required for nitrate respiration and persistence under hypoxic conditions. Moreover, the results obtained in *M*. *kansasii* suggest that the acquisition of *moaA1-D1* represented a key step during the course of evolution of the MTBC ancestor in becoming adapted to hypoxia.

### The *moaA1-D1* locus sustains *Mtb* entry into dormancy

**Fig 3:**
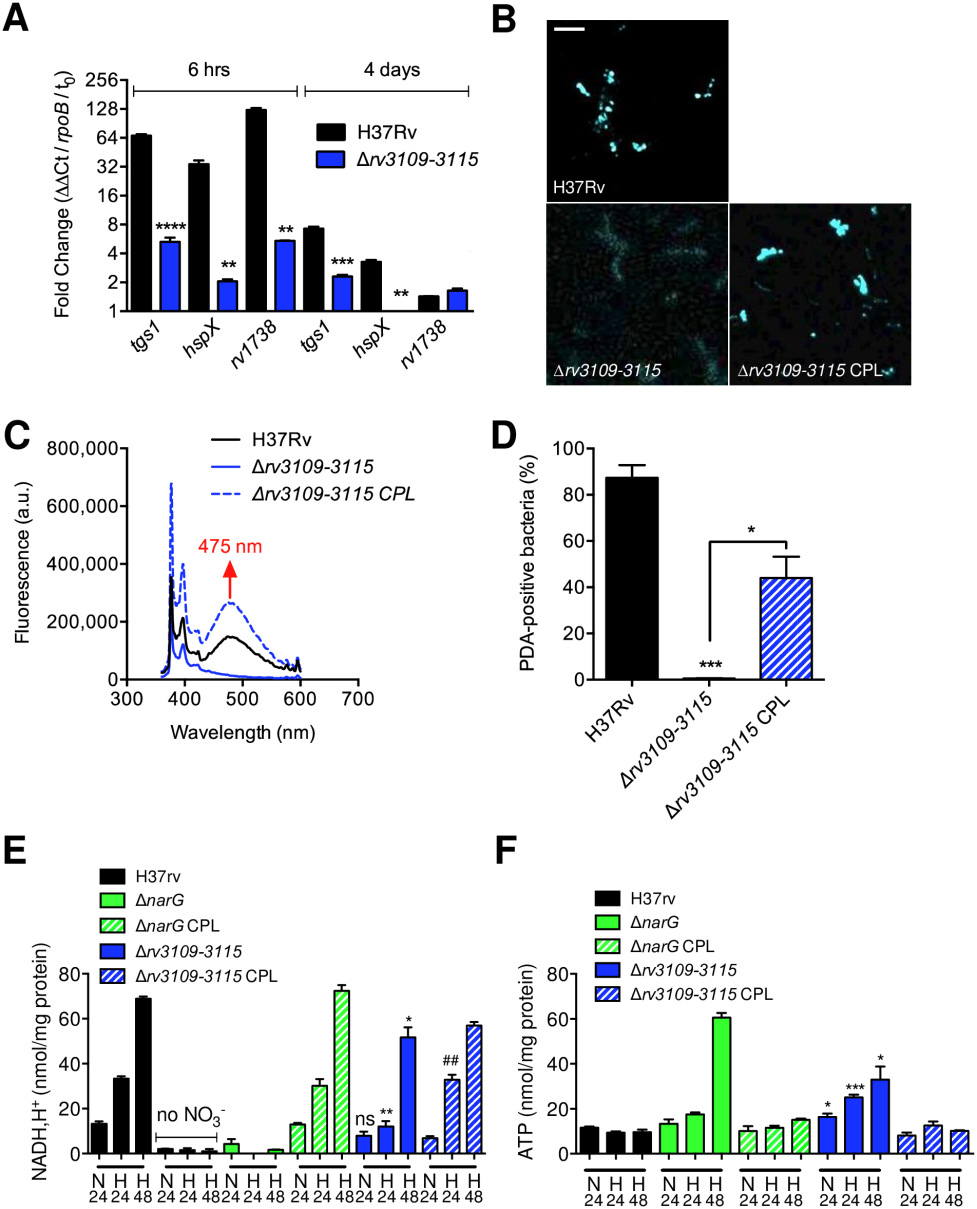
The *moaA1-D1* locus is required for *Mtb* entry into dormancy`. (A) RT-qPCR quantification of expression of genes from the DosR regulon in bacteria cultivated for 6 h or 4 days in Sauton’s modified minimal medium after fast O_2_ depletion. Data show mean±s.e.m. of technical triplicates and are representative of 5 independent experiments. Statistical analysis was performed using the Student’s *t* test. **P < 0.01; ***P < 0.001; ****P < 0.0001. (B to D) Bacteria were grown for 30 days in the Wayne model in the presence of a fluorescently labelled fatty acid (PDA). After incorporation of PDA in TAG, and TAG accumulation in the bacteria, lipid droplet formation was visualized by confocal microscopy (B). Specific accumulation of PDA into TAG was confirmed by fluorescence spectrometry (C) and thin layer chromatrography (S6A Fig). PDA accumulation in bacteria was quantified by flow cytometry (D, S6B Fig). In (B), bar represents 5 μm. In (D), data show mean±s.e.m. of biological replicates (n = 3). Each biological replicate was measured in technical triplicates and statistical analyses were performed on the summary of all the biological replicates. Data were analyzed using the Student’s *t*-test; *P < 0.05; ***P < 0.001. (E and F) Bacteria were grown in Sauton’s modified minimal medium. After 24 h in normoxia (N24), or 24 (H24) or 48 (H48) h after fast O_2_ depletion, bacteria were lysed for quantification of intracellular [NADH,H^+^] (E) or intracellular [ATP] (F). Data show mean±s.e.m. of biological replicates (n = 3). Each biological replicate was measured in technical triplicates and statistical analyses were performed on the summary of all the biological replicates. The graphs are representative of at least 3 independent experiments. Statistical analysis was performed using the Student’s *t* test. *P < 0.05; ***P < 0.001; ^##^*P*<0.01. * indicates comparison between the Δ*rv3109-15* mutant and the WT strain; ^#^ indicates comparison between the Δ*rv3109-15* mutant and the complemented strain. The graph is representative of 3 independent experiments. In all panels were the complemented strain is displayed, the I528 cosmid was used for complementation.

Because early adaptation to hypoxia is a key step in mycobacterial entry into dormancy, we next asked whether the *moaA1-D1* locus played a part in this process. We measured expression of three dormancy-related genes of the so-called DosR regulon [29,30], namely *tgs1*, *hspX*/*acr* and *rv1738*, in the wild type and Δ*rv3109-15* mutant at early (6 h) or late (4 days) stages after O_2_ depletion. At both stages induction of the DosR-regulated genes was impaired in the mutant strain (Fig 3A). As a consequence of impaired induction of *tgs1*, accumulation of triacylglycerol (TAG), which is a hallmark of mycobacterial dormancy [31], was also found impaired in the Δ*rv3109-15* mutant and restored in the complemented strain (Fig 3B–3D, S6 Fig). Mycobacterial entry into dormancy is associated with a modification in the redox status of the cell, which is accompanied by modifications in ATP turnover. In our model, incubation of the Δ*rv3109-15* mutant in hypoxic conditions resulted in a diminished generation of reduced NAD (Fig 3E) and an increased production of ATP (Fig 3F), compared to the wild type and complemented strains. As controls, the wild type strain cultivated in the absence of nitrate or a *Mtb* mutant inactivated in *narG* [32] (Δ*narG*), which encodes the catalytic subunit of the membrane-bound NR NarGHI, were both unable to produce reduced NAD, and over-produced ATP in the same conditions (Fig 3E and 3F).

Altogether, these findings indicate that the *moaA1-D1* inactivation partially phenocopies *narG* deletion, and that the *moaA1-D1* locus is involved in *Mtb* adaptation and proper entry into dormancy.

### The MoaR1 transcriptional regulator is hypoxia-responsive, induces expression of *moaA1-D1*, and sustains *Mtb* nitrate reduction and entry into dormancy

**Fig 4:**
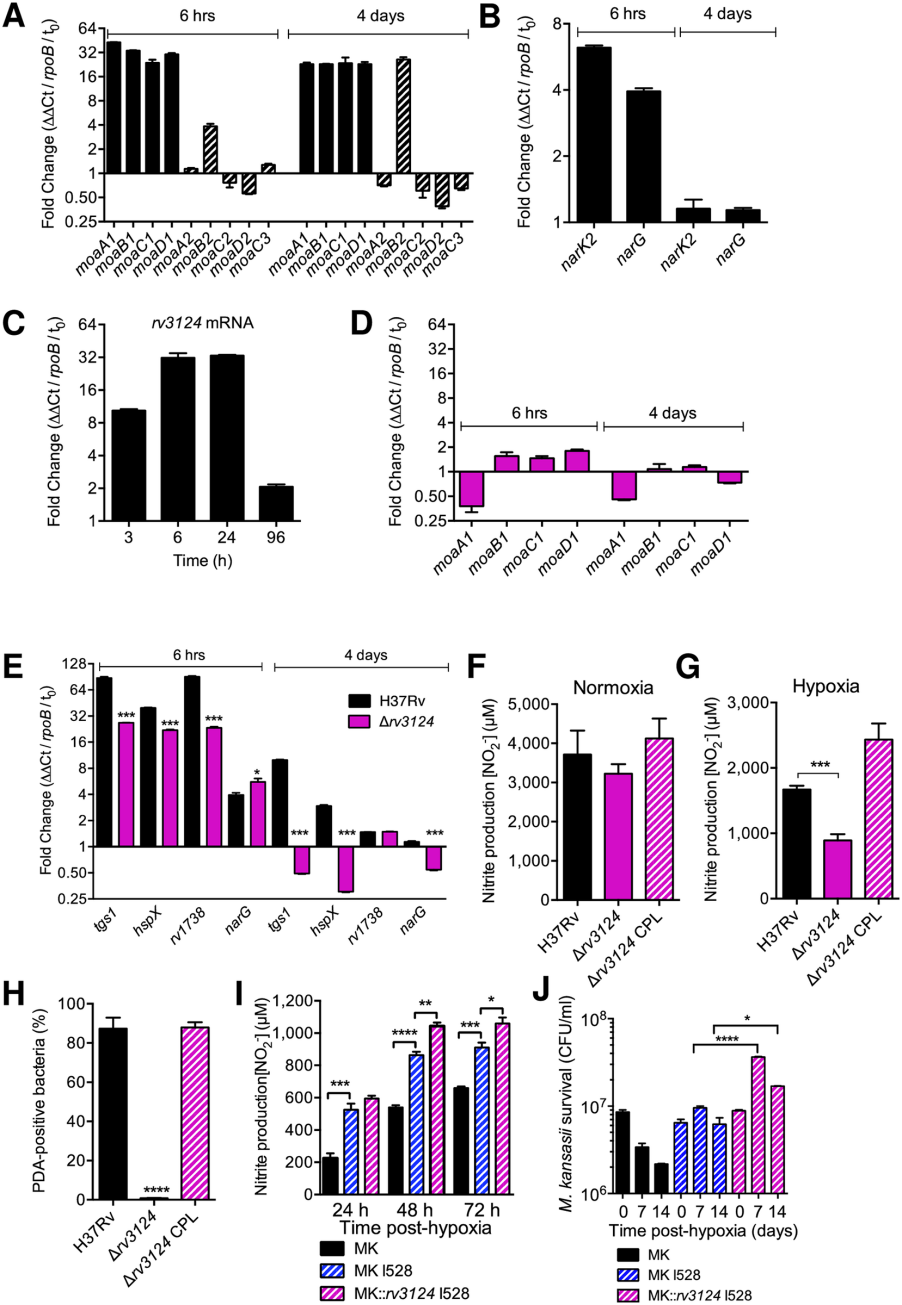
MoaR1/Rv3124 controls *Mtb* nitrate respiration and entry into dormancy in hypoxic conditions. (A to C) RT-qPCR quantification of expression of *Mtb moa* genes (A), genes involved in nitrate transport and utilization (B), and the *moaR1/rv3124* regulator (C) in H37Rv (wild-type). (D and E) RT-qPCR quantification of expression of *moA* genes (D) and DosR-dependent genes (E) in mutant strain inactivated in *moaR1* (D,E) and in H37Rv (E). In (A-E), bacteria were cultivated in Sauton’s modified minimal medium for the indicated time periods after fast O_2_ depletion before RNA extraction. In (A), solid bars indicate genes of the *moaA1-D1* locus, hatched bars represent other *moa* genes. Data show mean±s.e.m. of technical triplicates and are representative of 5 independent experiments. In (E), statistical analysis was performed using the Student’s *t* test. *P<0.05; **P < 0.01; ***P < 0.001. Comparison is between the Δ*rv3124* mutant and the WT strain. (F to H) Nitrate utilization (F and G) and PDA accumulation in bacteria (H) cultivated in Sauton’s modified minimal medium under normoxic (F) or 14 days after fast O_2_ depletion (G), or 30 days in the Wayne model (H). In (H), PDA accumulation was measured as in Fig 3D. (I) Nitrate utilization by *M*. *kansasii* (MK) or recombinant MK strains harboring the I528 cosmid with or without a *moaR1*/*rv3124*-encoding plasmid under hypoxia. (J) Survival of *M*. *kansasii* (MK) or recombinant MK strains harboring the I528 cosmid with or without a *moaR1*/*rv3124*-encoding plasmid under hypoxia. In panels (F-H), data show mean±s.e.m. of biological replicates (n = 3–4). Each biological replicate was measured in technical triplicates and statistical analyses were performed on the summary of all the biological replicates. The graphs are representative of at least 3 independent experiments. Statistical analysis was performed using the Student’s *t* test. *P < 0.05; **P<0.01; ***P < 0.001; ****P<0.0001.

Because the *Mtb* H37Rv genome harbors paralogues of *moaA1-D1* genes, namely *moaA2-D2* and *moaC3*, we wondered whether hypoxia could act as a triggering signal for expression of all or a subset of these genes. Strikingly, we found that while expression of *moaA1-D1* was strongly upregulated by hypoxia, expression of all their homologues but *moaB2* was irresponsive to hypoxia (Fig 4A). At a first glance, these data are different to results of previous studies, in which hypoxic stress was not associated with induction of the *moaA1-D1* locus [29,33]. However in these previous studies, bacteria were cultivated in a rich medium (*i*.*e*. 7H9/ADC). In our hands also, hypoxic stress in this rich medium barely induced *moaA1-D1* expression (S7A Fig). This indicates that the *moaA1-D1* locus is hypoxia-responsive when bacteria are cultivated in minimal medium only, which mimics the nutrient-poor conditions found in granulomas *in vivo*. At an early stage during the transition to hypoxia, we also found that expression of the nitrate transporter NarK2-, and the NR catalytic subunit NarG-encoding genes was induced (Fig 4B). This confirms previous findings obtained for NarK2 [21]. Regarding NarG, our findings are reminiscent of the results of a recent study in which *narG* expression was induced after addition of nitrate in hypoxic conditions [34].

The MoaR1 (Rv3124) transcriptional regulator, which is encoded by a gene in the Moco-1 genomic island, was shown to activate *moaA1-D1* gene expression in recombinant Rv3124-overexpressing *Mtb* and *Mycobacterium bovis* bacillus Calmette Guérin (BCG) strains [35]. Here, we found that *moaR1* expression was also induced by hypoxia (Fig 4C). Again, such induction was only observed when bacteria were cultivated in minimal medium, and not in rich medium (S7B Fig). Strikingly, hypoxic induction of *moaA1-D1* was abolished in a *Mtb* mutant strain deleted of the *moaR1* gene (Δ*rv3124* mutant), at both early (6 h) and late (4 days) stages after transition to hypoxia (Fig 4D). As observed in the Δ*rv3109-15* mutant, the *moaR1*-deleted mutant was likewise impaired in induction of the dormancy genes *tgs1*, *hspX*/*acr*, *rv1738* (Fig 4E), reflecting an altered entry into dormancy. Similarly, the Δ*rv3124* mutant was impaired in nitrate reduction and TAG accumulation in hypoxic, but not normoxic conditions (Fig 4F–4H). Finally, heterologous overexpression of *moaR1*/*rv3124* in a *moaA1-D1*-complemented recombinant *M*. *kansasii* strain, whose genome does not contain a *moaR1* homologue, resulted in increased nitrate reduction (Fig 4I), and further enabled this bacterium to survive hypoxia (Fig 4J). Altogether, these data show that MoaR1 is a hypoxia-responsive transcriptional activator that drives *moaA1-D1* expression and sustains *Mtb* nitrate reduction and entry into dormancy in hypoxic and nutrient-scarce conditions.

#### The *moaA1-D1* locus is required for *Mtb* growth and survival in hypoxic lesions *in vivo*

**Fig 5:**
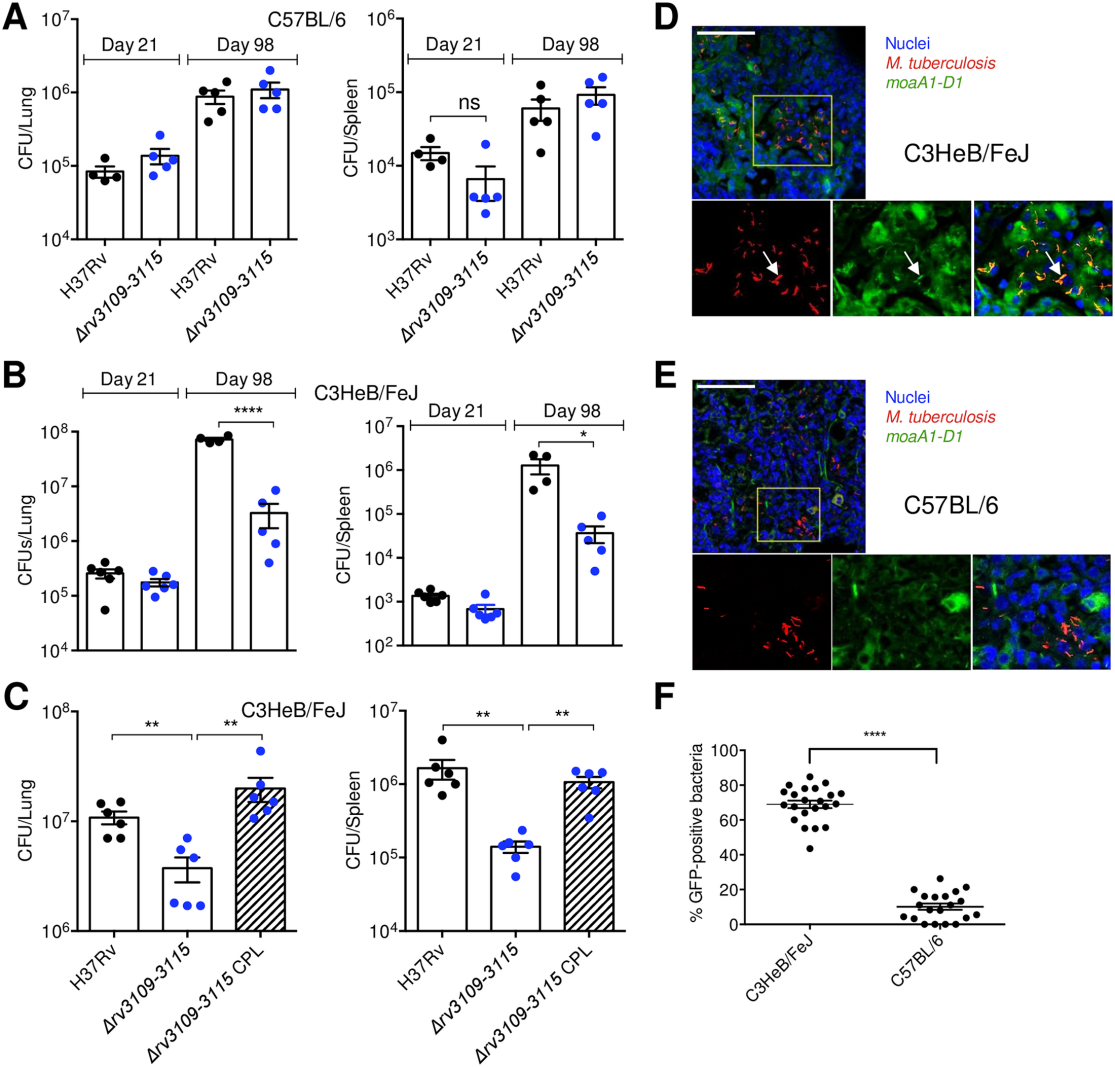
The *moaA1-D1* locus is required for persistence of *Mtb in vivo* in hypoxic granulomas. (A to C) C57BL/6 (A) or C3HeB/FeJ (B,C) mice were infected intranasally with ≈200 CFUs of the indicated bacterial strains. Lungs (left panels) and spleen (right panels) were collected after 21 (A and B) or 98 (A to C) days post-infection and analyzed for colony-forming unit (CFU) content. Statistical analysis was performed using the Student’s *t* test. *P < 0.05, **P < 0.01; ****P < 0.0001. (D and E) C3HeB/FeJ (D) or C57BL/6 (E) mice were infected with ≈200 CFUs of the red fluorescent protein-expressing reporter strain of *Mtb* encoding GFP under the control of the *moaA1-D1* promoter region. Cell nuclei were labeled with TOPRO-3 (blue). Bars represent 50 μm. (F) % of GFP-expressing bacteria in >20 lung lesions in *Mtb*-infected C3HeB/FeJ and C57BL/6 at day 98 post-infection. Lesions were sampled from 4 animals. Data show mean±s.e.m. and were analyzed using the Student’s *t* test. ****P<0.0001.

Based on the results above, we hypothesized that the *moaA1-D1* locus might play a part in the ability of the pathogen to survive hypoxia in lung lesions *in vivo*. To address this hypothesis, we subjected C57BL/6 mice, whose TB lung granulomas are not hypoxic, and C3HeB/FeJ mice that show hypoxic and necrotic lesions during *Mtb* infection [36–39], to intranasal infection with *Mtb* wild type, the Δ*rv3109-15* mutant or its complemented variant. We scored CFUs in the lungs and spleen, which reflects bacterial dissemination, of the infected animals at early (21 days) and late (98 days) stages of infection. Strikingly, while no growth defect was observed for the mutant strain in C57BL/6 mice (Fig 5A), the mutant was severely impaired (≈1.5 Log_10_) in lung colonization in C3HeB/FeJ mice (Fig 5B); this phenotype was observed at late time-points only, when at least a fraction of granulomas are known to be hypoxic [37,40], and could be reversed by genetic complementation (Fig 5C). Using a *Mtb* fluorescent reporter strain in which red fluorescent protein expression is constitutive and the *moaA1-D1* promoter region drives expression of GFP, we found that the *moaA1-D1* locus is induced *in vivo* in C3HeB/FeJ, as attested by the increased presence of yellow bacilli in lung lesions (Fig 5D), as compared to in C57BL/6 animals (Fig 5E and 5F). These findings indicate that induction of the *moaA1-D1* locus occurs in physiological conditions encountered *in vivo* and is required for proper lung colonization by *Mtb* in hypoxic lung lesions.

## Discussion

Our results show that the outstanding ability of *Mtb* to survive in hypoxic lung lesions is mediated, at least in part, by hypoxia-driven expression of MoaR1 and subsequent induction of the *moaA1-D1* locus, which is embedded in the horizontally acquired Moco-1 genomic island. Our results in C3HeB/FeJ mice are consistent with the previously reported impaired ability of a *Mtb mobA*-deficient mutant (S1 Fig) to persist in the lungs of guinea pigs, another animal model in which TB granulomas are hypoxic [41]. *In vitro*, our data show that the *moaA1-D1* locus governs improved nitrate respiration, survival to hypoxic stress and entry into dormancy under hypoxic and nutrient-scarce conditions.

### Regulation of the moaA1-D1 locus locus during Mtb adaptation to hypoxia

Our results highlight an important piece in the complex process of *Mtb* adaptation to hypoxia [42]. In this pathogen, early (<2 h) response to O_2_ depletion mostly relies on the DosR transcriptional regulator that is activated by its cognate redox- and O_2_- sensor histidine kinases, DosS and DosT. During prolonged hypoxic stress, the DosR-independent, so-called enduring hypoxic response (EHR) allows sustained bacterial survival in the absence of O_2_ and non-replicating persistence [43]. A recent system-level analysis revealed a complex network of transcriptional regulators involved in gene expression rewiring during *Mtb* transition into hypoxia or reaeration [44]. This network is centered on the transcription factor Rv0081 that connects the early (DosR) and late (EHR) responses to various metabolic switches that occur during mycobacterial adaptation to O_2_ depletion. Where does the MoaR1 regulation of the *moaA1-D1* locus stand in this network? Analyses of the global transcriptional effects of overexpression of >200 transcription factors [45] indicate that in addition to *moaA1-D1*, MoaR1 strongly activates expression of *rv3113*, *rv3114*, *rv3121* (*cyp141*) and *rv3125c* (*ppe49*), but is unlikely to modulate expression of any other genes in the *Mtb* genome. This suggests that MoaR1 is a Moco-1-specific regulator. The same study indicates that *moaR1* expression is slightly induced by other transcription factors, namely the hypoxia regulatory hub Rv0081 [44], the cell division-associated regulator Rv3260c/WhiB2, and the starvation-induced regulator Rv3291c. Moreover, in addition to MoaR1, the *moaA1-D1* locus can be activated by Rv0081, by Rv0324, a regulator involved in the HER [43], by Rv3286c/SigF, and by the histone-like protein Rv3597c/Lsr2. Although these data must be taken with caution as they were obtained using recombinant over-expressing systems, they nevertheless suggest that genes of the Moco-1 genomic island might also be under the control of one or more transcriptional regulators of the *Mtb* core genome (e.g. Rv0081), either directly or, more likely, after early induction of MoaR1; indeed our data show that genetic inactivation of MoaR1 completely abrogates hypoxia-mediated induction of the *moaA1-D1* locus. This complex regulatory network of Moco-1 gene expression during hypoxic stress will need to be further deciphered. In normoxia, basal expression of *moa* genes might be sufficient for proper Moco synthesis and normal function of the NR and other Moco-dependent enzymes [17], as has been shown for *moaD2* and the *moaD-E* hybrid gene *moaX* [23]. The differential responses of the various *moa* homologues presumably reflect *Mtb* adaptation to various oxygen-limited microenvironments encountered during the course of infection. Importantly, our study was conducted in the genetic background of the *Mtb* H37Rv strain in which the moa3 locus has been partially deleted. The phenotype of a *rv3109-12*-deleted mutant might be different in a background with an intact moa3 locus, such as *M*. *bovis*, which will need further investigation.

### Contribution of HGT to Mtb pathoadaptation

Our findings also bring new insights into the mechanisms that favored the emergence of *Mtb* as an extremely “successful” and widespread pathogen, and on the contribution of ancient HGT events to this process, which has long been disregarded. The presence of the Moco-1 locus, either in full or partially deleted, in *M*. *canettii* strains representing very early branching lineages of the tubercle bacilli [14], indicates that this region was acquired at an early time point in the evolution of *Mtb*, after the divergence from non-tuberculous mycobacteria but before clonal expansion of the MTBC. The presence of Moco-1 in the *M*. *canettii* genomes also offers a plausible explanation for the putative environmental origin of this region inferred from our genomic signature analysis. While the MTBC represents obligate mammalian-adapted pathogens, several lines of evidence suggest that *M*. *canettii* is less adapted to mammalian hosts and might have an environmental reservoir [8,14,46,47]. The observed interruption of the *moaA1-D1* gene set in some *M*. *canettii* strains, might explain, at least in part, why these bacteria are less adapted to mammalian hosts than *Mtb*. Such a reservoir might have favored direct contacts and hence HGT between the ancestral *M*. *canettii*-like pool and environmental bacteria, such as *Burkholderia* spp. or other close species. Our results evoke that lateral acquisition of the *moaA1-D1* locus and the MoaR1 transcriptional regulator by such (an) ancestor(s) improved its/their potential to rapidly adapt its/their metabolism to nitrate respiration under hypoxia, which is a hallmark of most TB granulomas. This scenario is also strongly supported by our findings that introduction and overexpression of *moaA1-D1* in the environmental mycobacterium *M*. *kansasii*, representing the phylogenetically closest non-tuberculosis mycobacterial species, enabled the latter recombinant strain to both greatly improve its nitrate respiration and readily survive upon oxygen depletion. Thus, several lines of evidence suggest that the unique ancestral acquisition of this locus represented a significant evolutionary shift towards improved metabolic adaptation of an environmental mycobacterial recipient species to stringent hypoxic conditions. Yet, the role of the *moaA1-D1* gene set in species of the MTBC that harbor a complete version of the *moaA3-X* paralogue locus, such as *Mycobacterium bovis*, remains to be explored. More generally, our findings raise intriguing questions about which of the up to 9% of the *Mtb* genes plausibly acquired by exogenous HGT [9,11,12] might have supported *Mtb* evolution towards pathogenicity.

### General conclusion

Taken together, our results thus suggest an evolutionary scenario in which the MTBC ancestor has progressively “learned” to become a most successful pathogen in part by gain of critical functions, through natural selection following exogenous HGT from its environmental neighbors, and by gene or gene allele shuttling within an ancestral *M*. *canettii*-like strain pools. Our recent data indicate that this adaptation was supplemented by parallel loss-of-function such as that affecting the *pks5* locus, which resulted in bacterial surface remodeling and increased virulence during the transition from a generalist *M*. *canettii*-like ancestor to the obligate pathogens of the MTBC [48]. On a more practical aspect, deciphering such critical steps in early *Mtb* pathoevolution may provide clues for identifying yet unknown vulnerable targets of the tubercle bacillus, as outlined in this study, which might be of interest for the development of novel therapeutic intervention against TB. In line with this, an inhibitor of MoeW, another horizontally acquired enzyme involved in Moco biosynthesis [9], was reported to improve *Mtb* killing [49]. A broader exploration of the potential of the many *Mtb* Moco biosynthesis enzymes, and those of the Moco-1 island in particular, as drug targets probably holds great promise for tackling TB, including latent infection.

## Materials and methods

### Mycobacteria and culture conditions

Mycobacteria were grown at 37°C in Middlebrook 7H9 medium (Difco) supplemented with 10% albumin-dextrose-catalase (ADC, Difco) and 0.05% Tyloxapol (Sigma), or on Middlebrook 7H11 agar medium (Difco) supplemented with 10% oleic acid-albumin-dextrose-catalase (OADC, Difco). When required, kanamycin, hygromycin or streptomycin were added to the culture media. In the Wayne model, bacteria were grown in Dubos medium (Difco) supplemented with 10% Dubos medium albumin (Difco) and 0.05% Tween 80 (Sigma) in glass tubes tightly closed with screwed caps and tight rubber caps, and incubated at 37°C, as previously described [28]. In experiments using nitrate as sole nitrogen source (fast hypoxia model), bacteria were first grown in Sauton’s modified medium containing 0.05% Tyloxapol, 0.5 g/L KH_2_PO_4_, 0.5 g/L MgSO_4_, 2 g/L citric acid, 10 g/L glycerol and 5g/L asparagine prepared in tap water and neutralized to pH 7.0 with NaOH. Before switching to hypoxia, bacteria were washed with PBS and asparagine was replaced with NaNO_3_ (10 mM). Anaerobic conditions were generated in an anaerobic jar containing a GasPak EZ anaerobe container system (Fisher scientific). Nitrite production in culture media was measured using the Griess reagent assay (Sigma). For measurement of intracellular [NADH, H^+^], bacteria were lysed at 65°C for 20 minutes and NADH,H^+^ was quantified with a NAD/NADH Quantitation Kit (Sigma). Total protein concentration was determined using the Micro BCA Protein Assay Kit (Thermo Scientific). Intracellular [ATP] was quantified after lysis of the bacteria for 10 minutes at 100°C using the BacTiter-Glo Microbial Cell Viability Assay (Promega).

### Construction of *Mtb* mutants and complemented strains

All mutant strains of *Mtb* were constructed by allelic exchange using the recombineering method [27]. Briefly, allelic exchange substrates for the Δ*rv3109-3115*, Δ*rv3115-3119* and Δ*mog* mutants, were prepared by amplifying from *Mtb* H37Rv genomic DNA two 1‐kb DNA fragments flanking the regions to be deleted that were inserted on each side of a kanamycin resistance cassette, into a pGem5Z plasmid. The recombination substrate was recovered by enzymatic digestion and purified from agarose gels. For the Δ*rv3124* strain, a DNA fragment of 1311 bp (with *rv3124* in position 442-1311bp) was amplified by PCR, cloned into pGem5Z and a kanamycin resistance cassette was inserted at position 388 bp of *rv3124*. Linearized constructs were introduced by electroporation in *Mtb* competent cells carrying pJV53H, a hygromycin resistant pJV53-derived plasmid expressing recombineering proteins. H37Rv::pJV53H was grown in 7H9-ADC-Tween 80 in the presence of hygromycin (50 μg/ml) until mid-log phase and expression of recombineering enzymes was induced by 0.2% acetamide (Sigma) overnight at 37°C. After induction, electrocompetent bacteria were prepared and electroporation performed with 100 ng of the linearized fragments. After 48 h incubation at 37°C in 7H9-ADC-Tween 80 without antibiotic, bacteria were plated onto 7H11-OADC agar medium in the presence of kanamycin (50 μg/ml). Kanamycin resistant clones were harvested, grown in 7H9-ADC-Tween 80 in the presence of kanamycin and verified to carry the expected allele replacement by PCR. The pJV53H plasmid was then lost by serial rounds of culture without antibiotic. For complementation of the Δ*rv3109-3115* strain, we used the pYUB412-derived integrative cosmid I528 [50]. I528 confers resistance to hygromycin and harbors a DNA fragment encompassing the region 3,454 to 3,485 kbp in the *Mtb* H37Rv genome. For complementation of the Δ*rv3115-3119* and Δ*rv3124* strains, we used the pYUB412-derived integrative cosmid IE240, which harbors a genomic fragment from *Mtb* Erdman corresponding to the genomic region 3,479 to 3,516 kbp of *Mtb* H37Rv. The description of the cosmids used in the study, together with that of the deleted regions in the mutants, are provided in S8 Fig. The Δ*mog* complemented strain was obtained after transformation with a pVV16*mog* vector with *mog* gene expression under the control of the constitutive *hsp60* promoter. The Δn*arG* mutant and its complemented strain were described earlier [19]. Primers used for PCR are listed in S1 Table.

### Construction of recombinant strains of *M*. *kansasii*

For constitutive expression of *moaA1-D1* in *M*. *kansasii*, the *rv3109-rv3112* region was amplified by PCR from *Mtb* H37Rv genomic DNA and a plasmid containing this region under the control of a constitutive promoter (P1) was constructed by multisite gateway recombination using the procedures detailed by Schnappinger *et al*. [51]. The resulting vector was transformed into *M*. *kansasii* for a stable integration at the *att* site of the L5 mycobacteriophage. The same approach was used for the generation of a plasmid constitutively expressing *rv3124*. This latter construct was used for the transformation of a strain of *M*. *kansasii* previously transformed with the pYUB412-derived integrative cosmid I528 (described above). Primers used for PCR are listed in S1 Table.

### Construction of a *Mtb* reporter strain

A reporter strain of *Mtb* was constructed by transformation of wt *Mtb* strain with a reporter plasmid constructed by multisite gateway recombination using the procedures detailed by Schnappinger *et al*. [51]. The construct contains three different modules: the first one is the orf encoding the RFP under the control of a constitutive promoter (P1), the second one is a 1 kb region spanning from -900 to +100 bp of the start codon of *rv3109* and the third module is an orf encoding the GFP. In this construct, GFP is expressed under the control of the *rv3109* promoter, as previously defined by Mendoza Lopez *et al*. [35]. When transformed in *Mtb*, the construct is stably integrated in single copy at the *att* site of the L5 mycobacteriophage.

### Gene expression analysis

Strains were adapted in culture medium containing NaNO_3_ (10 mM) one week before they were incubated in hypoxic conditions. Total RNA was extracted from cultures grown to logarithmic phase (OD_600_ between 0.5 and 0.6) using the RNeasy kit (Qiagen) following manufacturer’s instructions with slight modifications. Briefly, 5 mL of culture were centrifuged for 10 min at 1,800x*g*, the pellet was resuspended in 700 μL of 0.1% β-mercaptoethanol-containing RLT lysis buffer, and 0.1 μm-diameter glass beads were added to the tubes. Cells were lysed by two 120-second pulses at full speed in a bead-beater device. The samples were centrifuged for 30 sec at 20,200x*g*. One volume of absolute ethanol was added to the filtrate, and total RNA purified with an RNeasy column following the manufacturer’s procedure. RNA samples were treated for 30 min with 2U of Turbo DNase (Turbo DNA *free* kit, Ambion). The amount and purity of RNA were quantified using a NanoDrop ND-1000 apparatus (Thermo Scientific) by measuring absorbance at 260/280 nm. Double-stranded cDNA was reverse-transcribed using the superscript III Reverse Trancriptase kit (Invitrogen), according to the manufacturer’s protocol. For real-time qPCR, specific primers (S1 Table) were designed using the QuantPrime software[52] and PCR reactions were performed using SYBR Green Premix Ex Taq (Ozyme), according to the manufacturer’s protocol. All real-time qPCR reactions were carried out using a 7500 Real-Time PCR System and data were analyzed using the 7500 Software version 2.3 (Applied Biosystems). PCR array data were calculated by the comparative cycle threshold method, normalized with *rpoB* housekeeping gene, and expressed as mean fold change in experimental samples relative to initial time. Primers used for Rt-qPCR are shown in S2 Table.

### Confocal microscopy, thin layer chromatography, fluorescence spectrometry & flow cytometry analysis of lipid accumulation in TAG

In order to visualize TAG accumulation, bacteria were cultivated for 30 days in Dubos-Tween-Albumin broth complemented with 50 μM of 1-pyrenedecanoic acid (PDA, Molecular Probes). Bacteria were harvested by centrifugation, fixed for 2 h with 4% paraformaldehyde (PFA) at room temperature, and examined by confocal laser scanning microscopy. Images were acquired with an LSM 710 microscope and recorded using the Zen software (Carl Zeiss, Inc.) with a λ_exc_ = 360 nm and a λ_em_ between 400-500nm. Images were analyzed using the ImageJ software. For thin layer chromatography analysis of lipid content, total lipids were extracted with CHCl_3_/MeOH (1/1) and separation was done on silica plates using a CHCl_3_/MeOH/H_2_0 60/16/2 mix as mobile phase. Fluorescence emission was recorded under UV illumination at λ_exc_ = 365 nm. Quantification of lipid droplets positive bacteria was performed in the same culture conditions; after PFA fixation, bacteria were analyzed by flow cytometry (λexc = 450 nm) using a LSR II flow cytometer (Becton Dickinson).

### Ethics statement

All animal experiments were performed in animal facilities that meet all legal requirements in France and by qualified personnel in such a way to minimize discomfort for the animals. All procedures including animal studies were conducted in strict accordance with French laws and regulations in compliance with the European community council directive 68/609/EEC guidelines and its implementation in France. All protocols were reviewed and approved by the Comité d’Ethique Midi-Pyrénées (reference MP/03/07/04/09).

### Mouse infection

Six- to eight-week-old female mice (C3HeB/FeJ, Jackson laboratory, or C57BL/6J, Charles River) were anesthetized with a cocktail of ketamine (60 mg/kg, Merial) and xylasine (10 mg/kg, Bayer) and infected intranasally with ≈200 CFUs of mycobacteria in 25 μL of PBS-0.01% Tween 80. At 21 and 98 days post-infection, mice were sacrificed, and lungs and spleen homogenates were plated onto 7H11 agar for CFU scoring. For histological analysis, mice were infected intranasally with ≈200 CFUs of the RFP- P*rv3109*-GFP reporter strain. Mice were sacrificed after 98 days infection and lungs and spleen were fixed for 24 hours at 4°C in Periodate-Lysine-Paraformaldehyde (PLP) prior freezing in Optimal cutting temperature compound (OCT). Ten μm-sections were realized using a cryostat before labelling with TOPRO-3 (Molecular Probes) and examination by confocal laser scanning microscopy.

### Statistics

Data were analyzed using the Student’s *t*-test (two-tailed).

### Bioinformatics analyses

The moa gene and protein sequences from *Mtb* (H37Rv), *M*. *bovis* (AF2122/97), *M*. *marinum* (M), *M*. *fortuitum* (ATCC 6841), *M*. *smegmatis* (MC^2^155), *M*. *avium* (104), *M*. *vaccae* (ATCC 25954), *M*. *abscessus* (ATCC 19977), *M*. *gilvum* (PYR-GCK), *M*. *kansasii* (ATCC 12478) and *M*. *gordonae* were retrieved and analyzed using the Ensembl Genomes interface [53].

Horizontal gene transfers were identified either based on the genomic signature and a Kullback-Leibler divergence [9,24], or from atypical codon usage [54]. Local Jensen-Shannon divergence in tetranucleotide frequency was calculated between sliding windows (5-kb in length, 100-bp step) over the *Mtb* genome and either itself or the pBVIE01 plasmid.

HGT donor candidates were screened using GOHTAM [25], which includes the signatures profiles, *i*.*e*. tetranucleotide frequencies, of all the species present in GenBank. The hit found for *rv3108-13* suggests that the most likely donor is closely related to the *Burkholderia vietnamiensis* G4 plasmids. Further investigations showed that the *B*. *vietnamiensis* plasmid pBVIE01 exhibits the closest genomic signature to that of the *rv3108-13* cluster. This hit exhibited a quality score of 4/5, from an Euclidean distance of 0.012, and a confidence score of 4/5 given the respective length of the sequences compared.

The genomic signature tree was built using GOHTAM by neighbor joining the pairwise comparison matrix of the 4-nucleotide word frequencies of all-versus-all taxa with an Euclidean distance [55]. The resulting distances and scale are subsequently converted into arbitrary units. Taxa are represented by either their whole chromosomal or whole specific plasmid sequence.

Similarity matrixes between homologous proteins were built from pairwise alignments with BLASTP (v 2.2.31+) [56] without sequence filters. The lower matrixes in orange depict the coverage of the alignment defined as the smallest aligned fraction of either the query or the subject sequence. The upper matrix depicts in blue-white-red the percentage of identity of the aligned fraction. Self-alignment of genes (diagonals) were not considered.

Alignments of the *moa* gene sequences and flanking regions were performed for *M*. *canettii* strains STB-A (CIPT 140010059), -D, -E, -G, -H, -I, -J, and -K and *Mtb* H37Rv, *M*. *bovis* AF2122/97, and *M*. *africanum* GM041182, using corresponding genome sequences as retrieved under Magnifying Genome (MaGe) server (https://www.genoscope.cns.fr/agc/microscope/home/index.php). Comparative alignments were performed based on analysis of gene synteny and BLAST searches, using a custom Multiple Annotation of Genomes and Differential Analysis (MAGDA) software, developed in the Plague and Yersinia pestis laboratory (Centre for Infection and Immunity of Lille/University of Lille). ACT comparison files were generated using MAUVE version 2.3.1 software to visualize the SNP densities in the *moa* gene sequences and flanking regions between STB-G and STB-I [57,58].

## Supporting information

S1 Fig Molybdenum cofactor biosynthesis in *E*. *coli* and *Mtb*. The pathway is adapted from Magalon & Mendel [16]. On the left side of the arrows are shown the enzymes involved in Moco synthesis in *E*. *coli*. On the right side are shown *Mtb* homologs of these enzymes, as retrieved by BLAST search. In blue are indicated the *Mtb* horizontally acquired homologs. MoaB and Mog proteins were shown to have MPT-adenylyl-transferase activity [60]. As such, MoaB1 was positioned at the same Moco biosynthetic step although its actual role has not yet been evaluated.
(PDF)

S2 Fig Genetic alignment of the MoCo-1 cluster from *Mtb* H37Rv, *M*. *bovis* AF2122/97, *M*. *africanum* GM041182 and nine *M*. *canettii* (alias STB) strains. Coding sequences of comparison strains are aligned relatively to their H37Rv reference counterparts, represented on top. Coding sequences in black and orange correspond to transposases and pseudogenes, respectively. Frameshifts are marked with step-shaped signs. Oblique red stripes indicate an incomplete coding sequence in STB-G due to probable genome assembly artifacts. H37Rv, *Mtb* H37Rv; *M*.*a* GM041182, *M*. *africanum* GM041182; *M*.*b* AF2122/97, *M*. *bovis* AF2122/97; STB-K, -I, -J, -G, -E, -L, -H, -A, -D, *M*. *canettii* of sequence types K, I, J, G, E, L, H, A and D [14].
(PDF)

S3 Fig SNP distribution between STB-G and STB-I aligned genome segments, showing a portion with a markedly lower SNP density that indicates a probable recombination region involving gene orthologs from *rv3105c* to *rv3117* between both strains. Red lines indicate individual SNPs identified between the compared genomes. Thicker or uneven red lines result from multiple SNPs in close proximity or shifts due to small indels. Predicted coding sequences are shown on both DNA strands of STB-G (top) and STB-I genomes (bottom), according to transcription to the right and left, respectively. The correspondence with the H37Rv gene orthologues is shown above the genome segment of STB-G. Dark gray boxes on the horizontal lines indicate a sequence contig break in STB-G interrupting the local genome alignment between both strains; genes downstream this local contig break are again aligned according to the synteny with H37Rv gene orthologues.
(PDF)

S4 Fig Blast-based similarity matrixes showing conservation of the Moa homologues. Lower half-matrixes depict the pairwise alignment coverage (fraction of the gene aligned, orange scale). Upper half-matrixes depict the alignment identity (blue-white-red scale). MoaD-E+X were displayed on the same plot to take the gene fusion in *moaX* into account. Lines and columns are the homologs in the same order. Auto-alignments (diagonals) are colored in grey. Considering the coverage values and the percentages of identity, the data indicate that: i/ MoaA1 clusters with MoaA3 but not with MoaA2, which clusters with the MoaA orthologs in non-TB mycobacteria, suggesting that MoaA1 and MoaA3 are more related and that given the identity values among the paralogs and orthologs outside the MTBC, one is a copy of the other; ii/ MoaB2 clusters with the MoaB orthologs outside the MTBC; iii/ MoaB1 clusters with MoaB3 but with an intermediate identity despite a good coverage, suggesting an accumulation of mutations or a different source; iv/ MoaC1 clusters with MoaC3 and to a lesser extend with MoaC2, which clusters with the MoaC orthologs outside the MTBC, suggesting that MoaC1 and MoaC3 are more related and that given the identity values among the paralogs and orthologs outside the MTBC, one is a copy of the other; v/ MoaD2 clusters with the MoaD orthologs outside the MTBC, but not with MoaD1; vi/ MoaE2 clusters with the MoaE orthologs outside the MTBC, but not with MoaE1; and vii/ MoaX matches both MoaD and MoaE orthologs with respective coverage, in line with the proposed origin of *moaX* as a fusion of the *moaD* and *moaE* genes.
(PDF)

S5 Fig Complementation of the Δ*rv3109-15* mutant with a *moaA1-D1*-encoding plasmid is equivalent to complementation with the I528 cosmid. Bacteria were cultivated for 14 days in modified Sauton’s minimal medium in hypoxic (after fast O_2_ depletion) conditions. Nitrate reduction was quantified by measuring nitrite production. For cosmid complementation, the I528 cosmid was used. For plasmid complementation, the moaA1-D1-encoding plasmid used in Fig 2F was used. Data show mean±s.e.m. of biological replicates (n = 4). Each biological replicate was measured in technical triplicates and the mean of the technical replicates were used for statistical analysis. Data were analyzed using the Student’s *t*-test; **P<0.01; NS, not significant. The graph is representative of 3 independent experiments.
(PDF)

S6 Fig Lack of TAG accumulation in the Δ*rv3109-3115* mutant strain, compared to the wild type or the complemented strain after cultivation for 30 days in the Wayne model, as measured by thin layer chromatography (A) or flow cytometry (B). In (A), total lipid extracts from the different strains were resolved on silica TLC using CHCl_3_/MeOH/H_2_O 60/16/2 mix as mobile phase. Fluorescence emission was recorded under UV illumination at λ_exc_ = 365 nm. Quantification of lipid droplet-positive bacteria was performed in the same culture conditions; after PFA fixation, bacteria were analyzed by flow cytometry at λ_exc_ = 450 nm.
(PDF)

S7 Fig The *Mtb moa* (A) and *moaR1* (B) genes are not induced in hypoxia in a nutrient-rich medium. *Mtb* H37Rv was cultivated in 7H9-ADC for the indicated periods of time after fast O_2_ depletion and before RNA extraction. Gene expression was quantified as in Fig 4. Data show mean±s.e.m. of technical duplicates and are representative of 3 independent experiments.
(PDF)

S8 Fig Organization of the genomic fragments inserted in the I528 (A) and IE240 (B) cosmids. The deleted regions in the Δ*rv3109-15* (A) and Δ*rv3115-19* (B) mutants are indicated in red.
(PDF)

S1 Table Primers used for RT-qPCR experiments. (DOCX)

S2 Table Primers used for PCR. (DOCX)
