# Supplementary material for: Horizontal acquisition of a hypoxia-responsive molybdenum cofactor biosynthesis pathway contributed to Mycobacterium tuberculosis pathoadaptation
Source: PLoS Pathog. 2017 Nov 27;13(11):e1006752. doi: 10.1371/journal.ppat.1006752 (PMC5720804; doi:10.1371/journal.ppat.1006752)
Supplement: S1 Fig — The pathway is adapted from Magalon & Mendel [16]. On the left side of the arrows are shown the enzymes involved in Moco synthesis in E. coli. On the right side are shown Mtb homologs of these enzymes, as retrieved by BLAST search. In blue are indicated the Mtb horizontally acquired homologs. MoaB and Mog proteins were shown to have MPT-adenylyl-transferase activity [60]. As such, MoaB1 was positioned at the same Moco biosynthetic step although its actual role has not yet been evaluated. (PDF) [file ppat.1006752.s001.pdf]

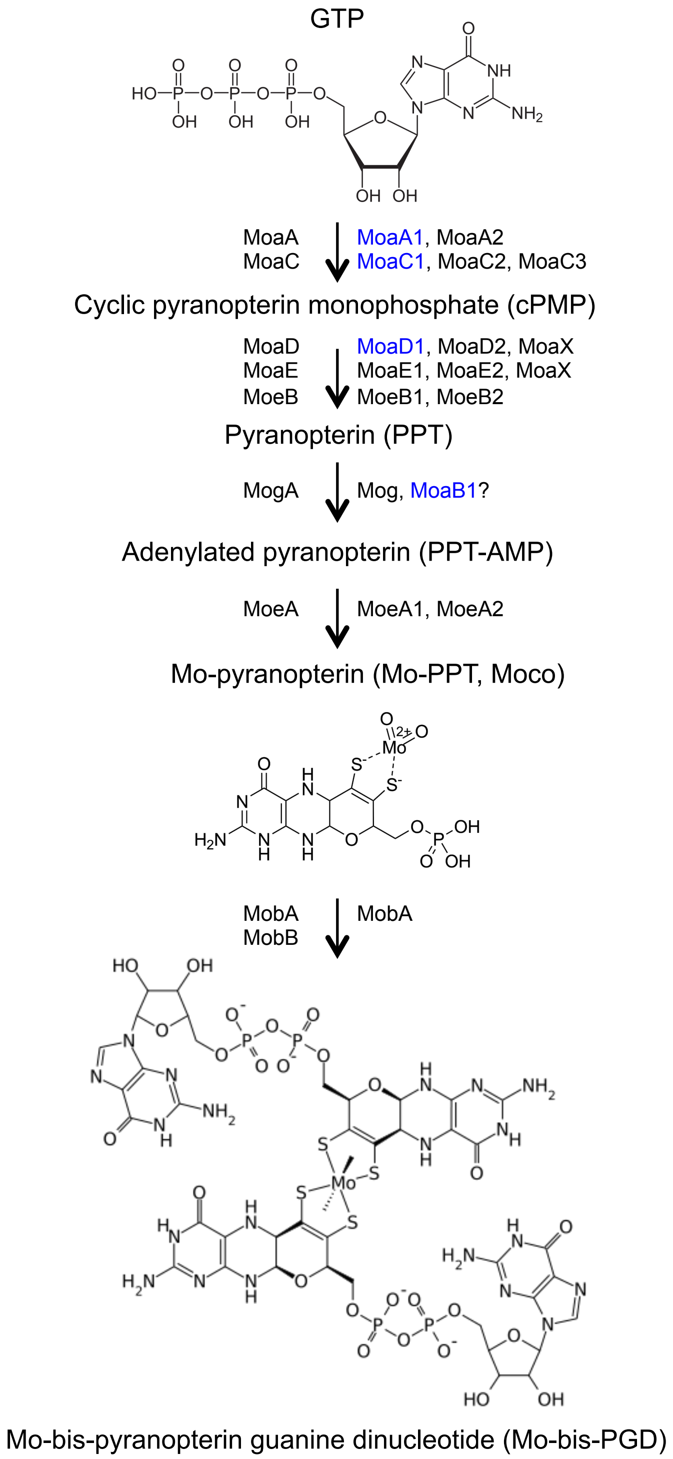

GTP
MoaA
MoaC
MoaA1, MoaA2
MoaC1, MoaC2, MoaC3
Cyclic pyranopterin monophosphate (cPMP)
MoaD
MoaE
MoeB
MoaD1, MoaD2, MoaX
MoaE1, MoaE2, MoaX
MoeB1, MoeB2
Pyranopterin (PPT)
MogA
Mog, MoaB1?
Adenylated pyranopterin (PPT-AMP)
MoeA
MoeA1, MoeA2
Mo-pyranopterin (Mo-PPT, Moco)
MobA
MobB
MobA
Mo-bis-pyranopterin guanine dinucleotide (Mo-bis-PGD)
