# Supplementary figures and images for: Horizontal acquisition of a hypoxia-responsive molybdenum cofactor biosynthesis pathway contributed to Mycobacterium tuberculosis pathoadaptation
Source: PLoS Pathog. 2017 Nov 27;13(11):e1006752. doi: 10.1371/journal.ppat.1006752 (PMC5720804; doi:10.1371/journal.ppat.1006752)

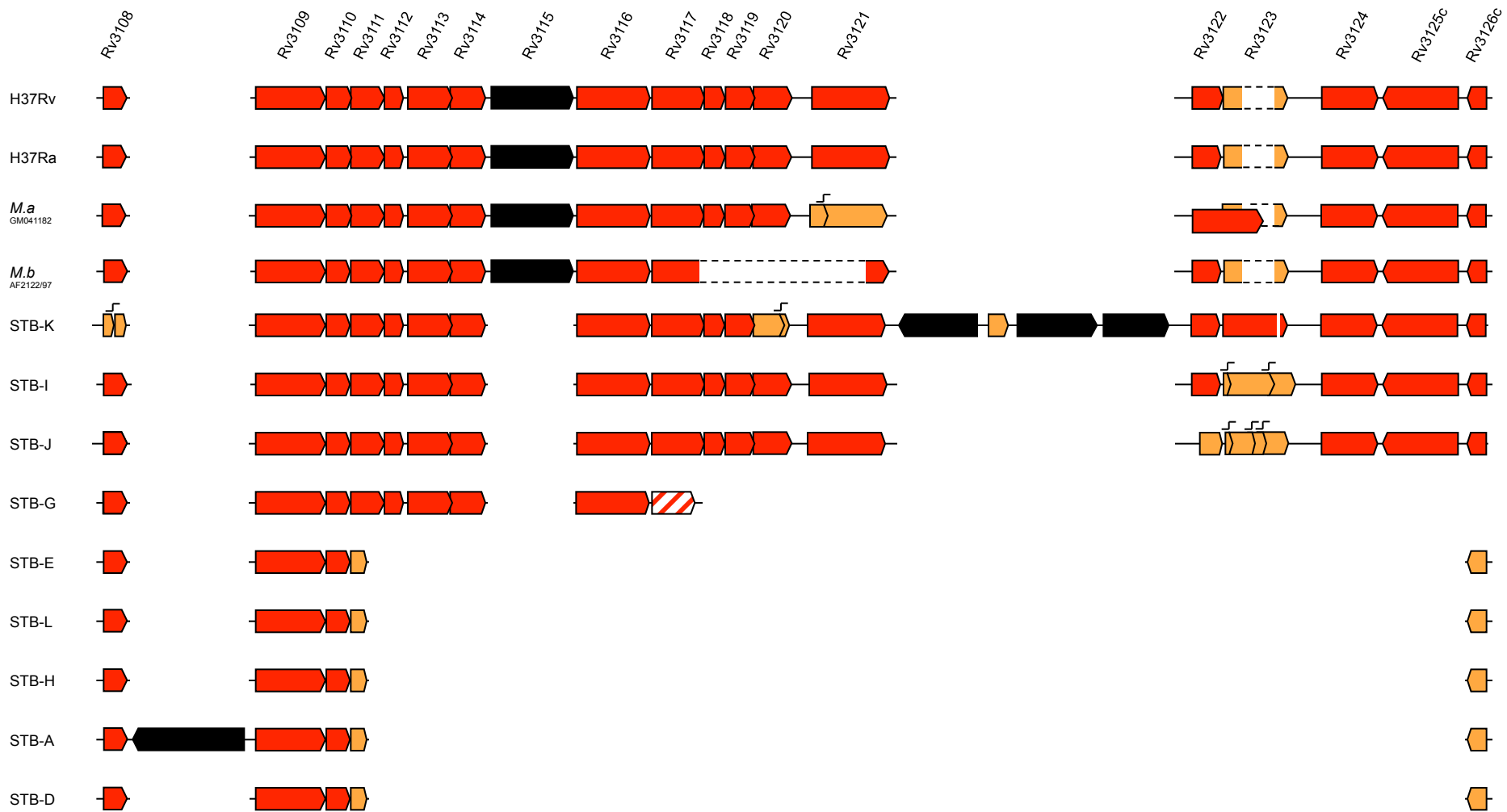

Supplement: S2 Fig — Coding sequences of comparison strains are aligned relatively to their H37Rv reference counterparts, represented on top. Coding sequences in black and orange correspond to transposases and pseudogenes, respectively. Frameshifts are marked with step-shaped signs. Oblique red stripes indicate an incomplete coding sequence in STB-G due to probable genome assembly artifacts. H37Rv, Mtb H37Rv; M.a GM041182, M. africanum GM041182; M.b AF2122/97, M. bovis AF2122/97; STB-K, -I, -J, -G, -E, -L, -H, -A, -D, M. canettii of sequence types K, I, J, G, E, L, H, A and D [14]. (PDF) [file ppat.1006752.s002.pdf]

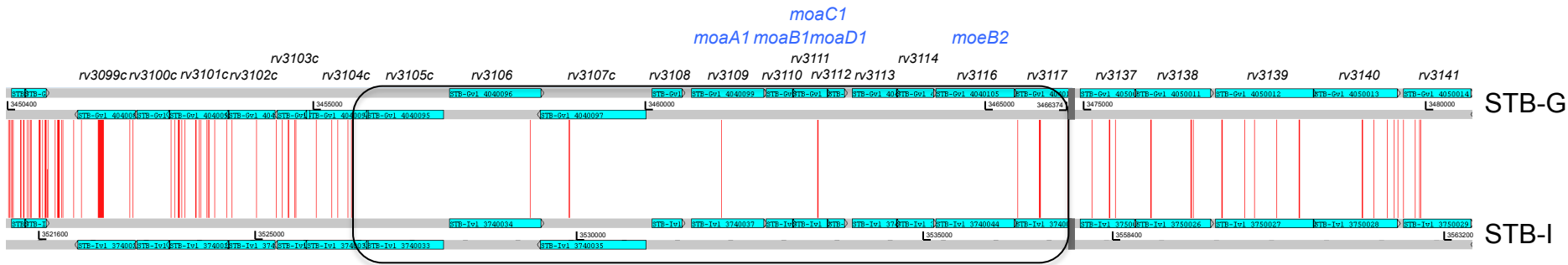

Likely inter-STB-G-STB-I recombination region

Supplement: S3 Fig — Red lines indicate individual SNPs identified between the compared genomes. Thicker or uneven red lines result from multiple SNPs in close proximity or shifts due to small indels. Predicted coding sequences are shown on both DNA strands of STB-G (top) and STB-I genomes (bottom), according to transcription to the right and left, respectively. The correspondence with the H37Rv gene orthologues is shown above the genome segment of STB-G. Dark gray boxes on the horizontal lines indicate a sequence contig break in STB-G interrupting the local genome alignment between both strains; genes downstream this local contig break are again aligned according to the synteny with H37Rv gene orthologues. (PDF) [file ppat.1006752.s003.pdf]

MoaA

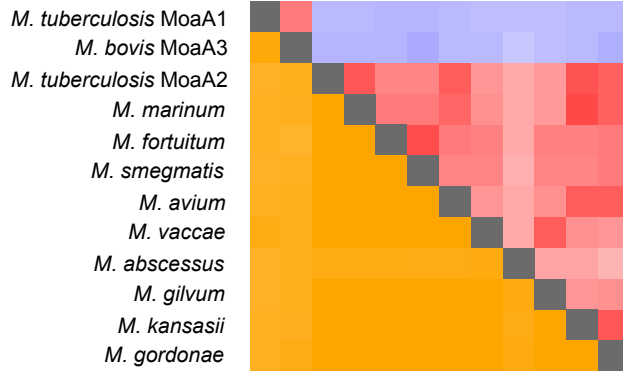

MoaB

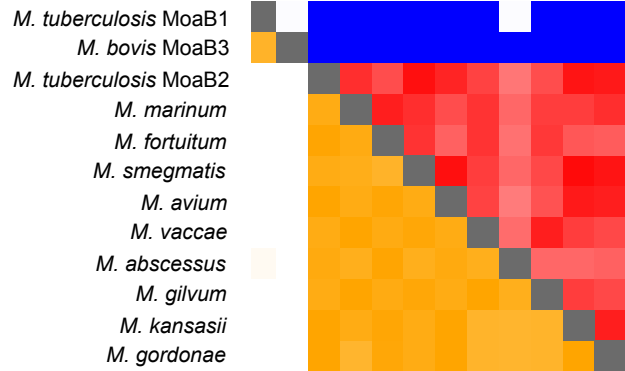

MoaC

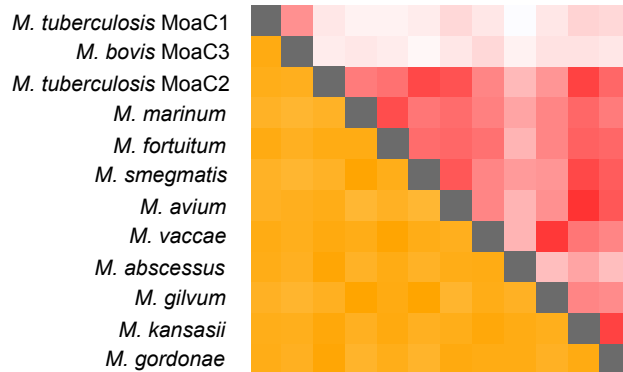

MoaD

MoaE

MoaX

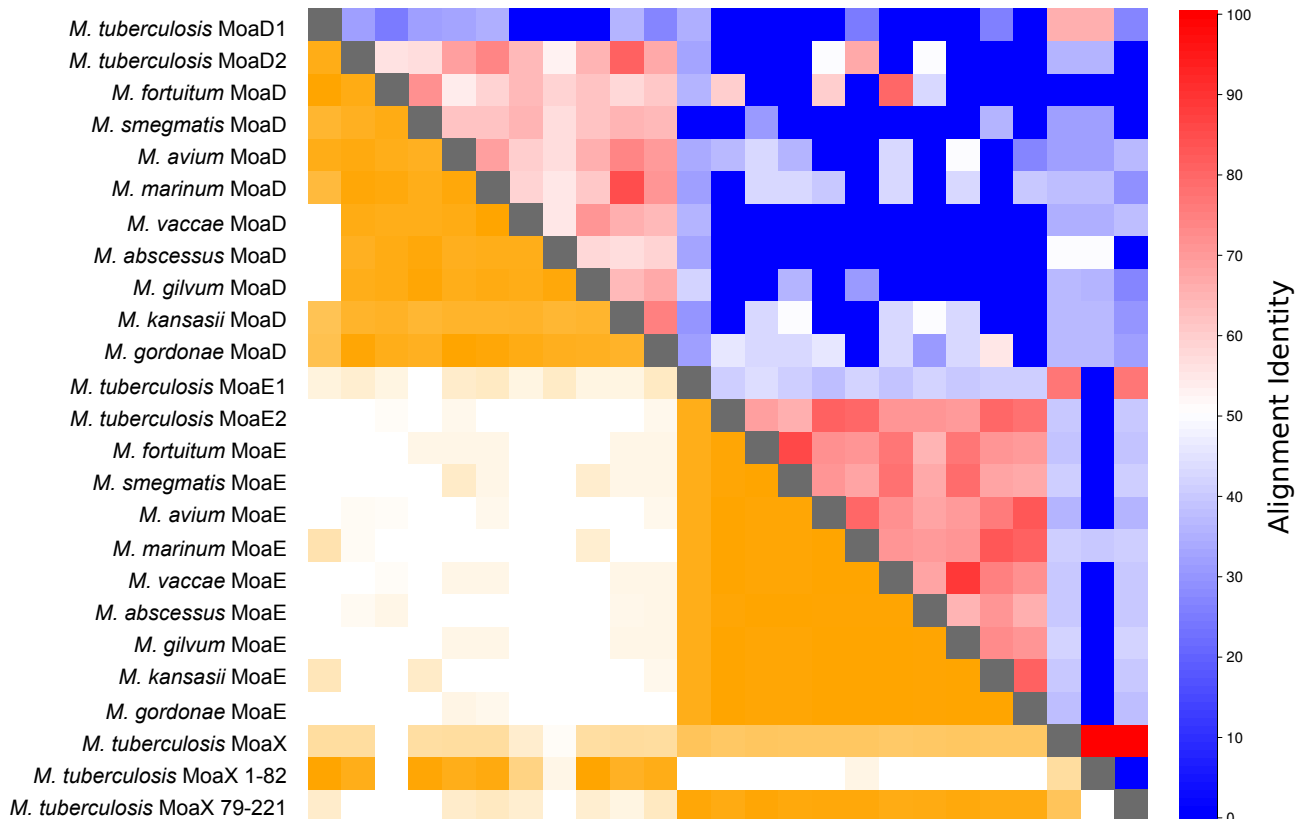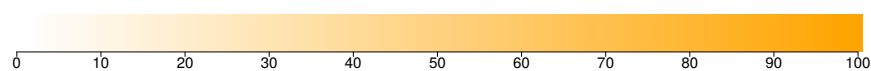

Alignment Coverage

Alignment Identity

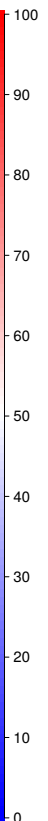

Supplement: S4 Fig — Lower half-matrixes depict the pairwise alignment coverage (fraction of the gene aligned, orange scale). Upper half-matrixes depict the alignment identity (blue-white-red scale). MoaD-E+X were displayed on the same plot to take the gene fusion in moaX into account. Lines and columns are the homologs in the same order. Auto-alignments (diagonals) are colored in grey. Considering the coverage values and the percentages of identity, the data indicate that: i/ MoaA1 clusters with MoaA3 but not with MoaA2, which clusters with the MoaA orthologs in non-TB mycobacteria, suggesting that MoaA1 and MoaA3 are more related and that given the identity values among the paralogs and orthologs outside the MTBC, one is a copy of the other; ii/ MoaB2 clusters with the MoaB orthologs outside the MTBC; iii/ MoaB1 clusters with MoaB3 but with an intermediate identity despite a good coverage, suggesting an accumulation of mutations or a different source; iv/ MoaC1 clusters with MoaC3 and to a lesser extend with MoaC2, which clusters with the MoaC orthologs outside the MTBC, suggesting that MoaC1 and MoaC3 are more related and that given the identity values among the paralogs and orthologs outside the MTBC, one is a copy of the other; v/ MoaD2 clusters with the MoaD orthologs outside the MTBC, but not with MoaD1; vi/ MoaE2 clusters with the MoaE orthologs outside the MTBC, but not with MoaE1; and vii/ MoaX matches both MoaD and MoaE orthologs with respective coverage, in line with the proposed origin of moaX as a fusion of the moaD and moaE genes. (PDF) [file ppat.1006752.s004.pdf]

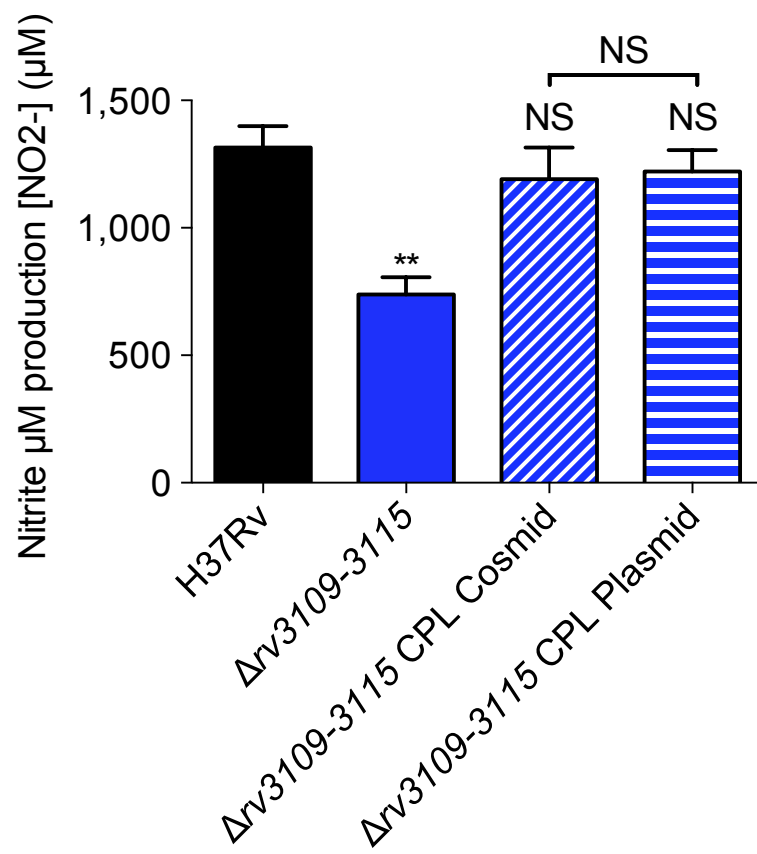

Supplement: S5 Fig — Bacteria were cultivated for 14 days in modified Sauton’s minimal medium in hypoxic (after fast O2 depletion) conditions. Nitrate reduction was quantified by measuring nitrite production. For cosmid complementation, the I528 cosmid was used. For plasmid complementation, the moaA1-D1-encoding plasmid used in Fig 2F was used. Data show mean±s.e.m. of biological replicates (n = 4). Each biological replicate was measured in technical triplicates and the mean of the technical replicates were used for statistical analysis. Data were analyzed using the Student’s t-test; **P<0.01; NS, not significant. The graph is representative of 3 independent experiments. (PDF) [file ppat.1006752.s005.pdf]

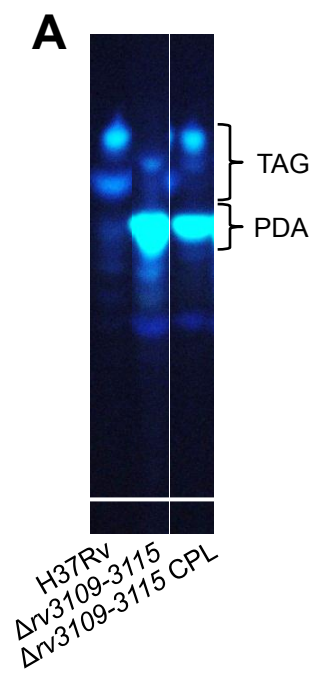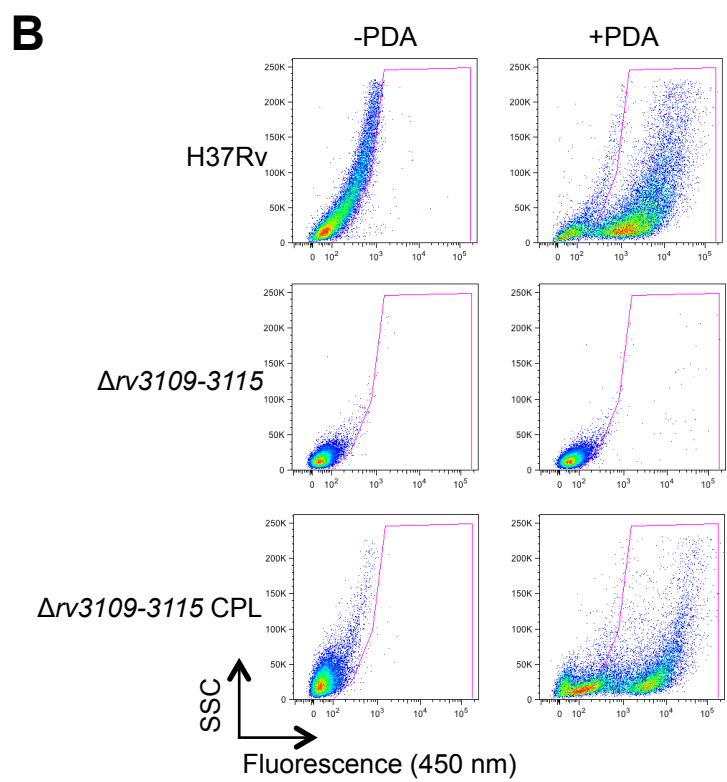

Supplement: S6 Fig — In (A), total lipid extracts from the different strains were resolved on silica TLC using CHCl3/MeOH/H2O 60/16/2 mix as mobile phase. Fluorescence emission was recorded under UV illumination at λexc = 365 nm. Quantification of lipid droplet-positive bacteria was performed in the same culture conditions; after PFA fixation, bacteria were analyzed by flow cytometry at λexc = 450 nm. (PDF) [file ppat.1006752.s006.pdf]

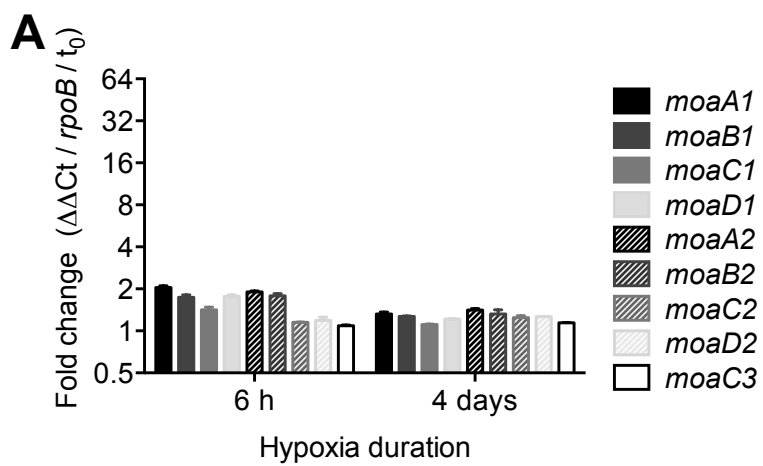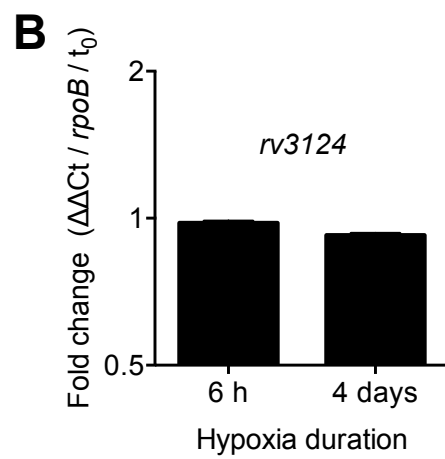

Supplement: S7 Fig — Mtb H37Rv was cultivated in 7H9-ADC for the indicated periods of time after fast O2 depletion and before RNA extraction. Gene expression was quantified as in Fig 4. Data show mean±s.e.m. of technical duplicates and are representative of 3 independent experiments. (PDF) [file ppat.1006752.s007.pdf]

**A**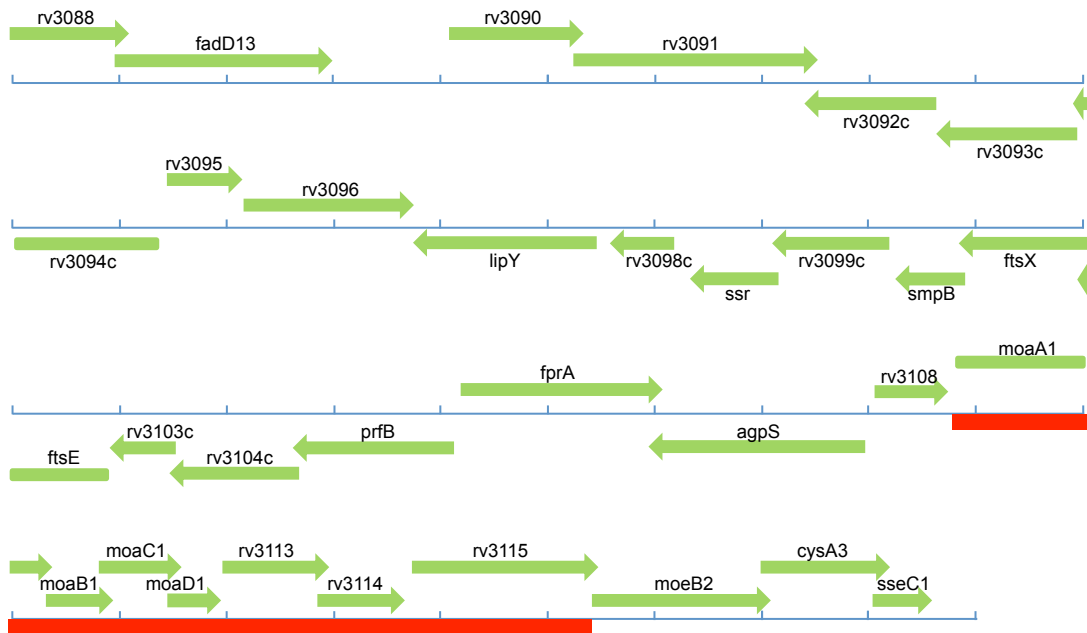**B**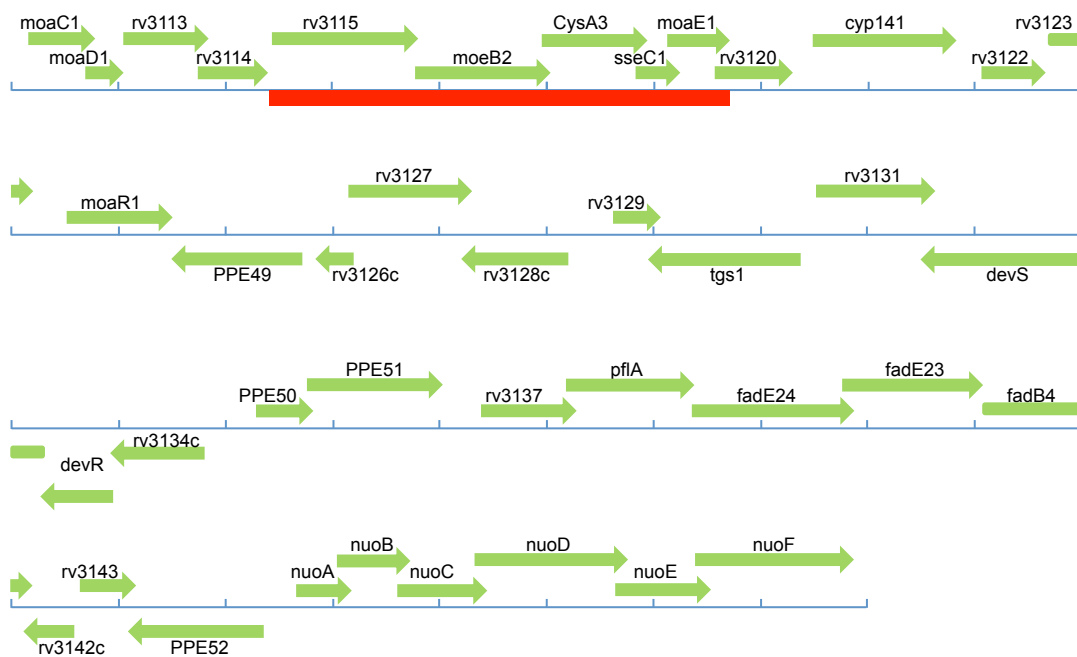

Supplement: S8 Fig — The deleted regions in the Δrv3109-15 (A) and Δrv3115-19 (B) mutants are indicated in red. (PDF) [file ppat.1006752.s008.pdf]
