# Supplementary material for: Horizontal acquisition of a hypoxia-responsive molybdenum cofactor biosynthesis pathway contributed to Mycobacterium tuberculosis pathoadaptation
Source: PLoS Pathog. 2017 Nov 27;13(11):e1006752. doi: 10.1371/journal.ppat.1006752 (PMC5720804; doi:10.1371/journal.ppat.1006752)
Supplement: S1 Table — (DOCX) [file ppat.1006752.s009.docx]

**S1 Table** Primers used for RT-qPCR experiments

| Gene | **Forward primer (5'-3')** | **Reverse primer (5'-3')** |
| --- | --- | --- |
| *rpoB* | TCGTTCTCTGACCCTCGTTTC | ACGTGCCCTTCTCGGTCATCA |
| *tgs1* | TCGCCACGGTGACAACAC | GGGACAGAGACAATTCGGAAATAC |
| *hspX* | CCGAGCGCACCGAGCAGAAG | GCCTTAATGTCGTCCTCGTCAGCA |
| *rv1738* | TGCGGCGACCAGTCGGATCA | GCCAGGCCAACACCCACCAATT |
| *narG* | GACAACACCAAGTTCGCCGACG | GCGCACATAGTCGACAAAGAACGG |
| *narK2* | AGGCGGATTGGGCGGTTAC | GGTGTAGGTACACGCGACCAG |
| *moaA1* | TGAGCCTTTGCCCAAACATG | AATGCAGCCATAAGCCATCG |
| *moaB1* | GGCTATCTGACCGACATCGAC | CATACATCGCCTCGACGGA |
| *moaC1* | CGTCAGGGCTCGTGATCAT | GGCACCGTCGGAAATCATC |
| *moaD1* | TGGCGGTCACCAATTCAT | CATCACCATCGCCGAGAAC |
| *moaA2* | gctgatacacatcgcggtaa | atgctatccagcgagacgtt |
| *moaB2* | aggtcgagatccgaaatgc | atctcgcggtccagaatg |
| *moaC2* | ttctggggcatccgactac | tcggtgatatcgaccatgtg |
| *moaD2* | CTGATAGACGGCCTGTCGGT | TCATCTCGGACGACAATCCC |
| *moaC3* | ACCGTGCCATGACGATCAC | CACCATCGCTGGGTTTGAC |
| *rv3124* | ctcattggagatcgacagca | aggagctcatcttcggtcaa |
