# Supplementary material for: Horizontal acquisition of a hypoxia-responsive molybdenum cofactor biosynthesis pathway contributed to Mycobacterium tuberculosis pathoadaptation
Source: PLoS Pathog. 2017 Nov 27;13(11):e1006752. doi: 10.1371/journal.ppat.1006752 (PMC5720804; doi:10.1371/journal.ppat.1006752)
Supplement: S2 Table — (DOCX) [file ppat.1006752.s010.docx]

**S2 Table** Primers used for PCR

| Primer | Forward primer (5'-3') | Reverse primer (5'-3') | Mutant strains |
| --- | --- | --- | --- |
| Δ*rv3109-15* left arm | CCATGGGTCATGGCCGGACACCAGT | GATATCCTACAATGGCGCTGATTTC | Δ*rv3109-15* |
| Δ*rv3109-15* right arm | CTGCAGCTTGGCCAGGAGGAGAGCA | GAGCTCGATTTCGCCTTTGGGTATC |  |
| Δ*rv3115-19* left arm | CATATGCCACCTTTCGCCGAATCTA | GTCGACTCAATGCGATGGATCGGTC | Δ*rv3115-19* |
| Δ*rv3115-19* right arm | GATATCGAGGGTGTCCGCGTTGCTG | CCATGGGCGAGCCCAACGTGCCCAT |  |
| Δ*rv3124* | CGAAGTGTGTGCATGAAACCCG | TTATGCCAAACCGATGGGAA | Δ*rv3124*/Δ*moaR1* |
| Δ*mog* left arm | GCATGCCAGCACCAGCAGTGCGTCG | ACTAGTGTGCTCATCGCCTCGTCCA | Δ*mog* |
| Δ*mog* right arm | CTGCAGCCGTTGGTGCGGCTGCGCA | CATATGTTAGGACTCGAACTCCGC |  |
| Δ*mog* CPL | CATATGAGCACCCGGTCCGC | AAGCTTTCATCGCGGGTGATC | Δ*mog* CPL |
| Δ*rv3124* CPL | CATATGCAATTCAACGTCTT | AAGCTTTTATGCCAAACCGA | Δ*rv3124* CPL |
